# Viscoelastic modelling and analysis of two-dimensional woven CNT-based multiscale fibre reinforced composite material system

**DOI:** 10.1186/s11671-024-04009-5

**Published:** 2024-04-30

**Authors:** Ashirbad Swain, Vignesh Palani, Sigil Francis, Benedict Thomas, Tarapada Roy

**Affiliations:** 1 School of Mechanical Engineering, Vellore Institute of Technology, Vellore, Tamil Nadu 632014 India; 2 Department of Mechanical Engineering, National Institute of Technology, Rourkela, Odisha 769008 India

**Keywords:** CNTs, Conventional carbon fibres, CNTs based woven fabric composite, Mori–Tanaka micromechanics, Weak viscoelastic interphase, Unit cell method, Strength of material method, Viscoelastic properties

## Abstract

Carbon nanotube (CNT) has fostered research as a promising nanomaterial for a variety of applications due to its exceptional mechanical, optical, and electrical characteristics. The present article proposes a novel and comprehensive micromechanical framework to assess the viscoelastic properties of a multiscale CNT-reinforced two-dimensional (2D) woven hybrid composite. It also focuses on demonstrating the utilisation of the proposed micromechanics in the dynamic analysis of shell structure. First, the detailed constructional attributes of the proposed trans-scale composite material system are described in detail. Then, according to the nature of the constructional feature, mathematical modelling of each constituent phase or building block’s material properties is established to evaluate the homogenised viscoelastic properties of the proposed composite material system. To highlight the novelty of this study, the viscoelastic characteristics of the modified matrix are developed using the micromechanics method of Mori–Tanaka (MT) in combination with the weak viscoelastic interphase (WI) theory. In the entire micromechanical framework, the CNTs are considered to be randomly oriented. The strength of the material (SOM) approach is used to establish mathematical frameworks for the viscoelastic characteristics of yarns, whereas the unit cell method (UCM) is used to determine the viscoelastic properties of the representative unit cell (RUC). Different numerical results have been obtained by varying the CNT composition, interface conditions, agglomeration, carbon fibre volume percentage, excitation frequency, and temperature. The influences of geometrical parameters like yarn thickness, width, and the gap length to yarn width ratio on the viscoelasticity of such composite material systems are also explored. The current study also addresses the issue of resultant anisotropic viscoelastic properties due to the use of dissimilar yarn thickness. The results of this micromechanical analysis provide valuable insights into the viscoelastic properties of the proposed composite material system and suggest its potential applications in vibration damping. To demonstrate the application of developed novel micromechanics in vibration analysis, as one of the main contributions, comprehensive numerical experiments are conducted on a shell panel. The results show a significant reduction in vibration amplitudes compared to traditional composite materials in the frequency response and transient response analyses. To focus on the aspect of micromechanical behaviour on dynamic response and for the purpose of brevity, only linear strain displacement relationships are considered for dynamic analysis. These insights could inform future research and development in the field of composite materials.

## Introduction

Woven fabric composites have been employed in a wide range of engineering applications. In woven composites, when a strand breaks in the weave, the load is carried by the surrounding fibres. However, in unidirectional composites, fibre breakage or puncture can affect strength over the entire length of the fibre. As a result, woven fabric composites have been a popular research topic for decades. Woven fabric in the composite material system offers improved overall strength and stiffness characteristics in all directions due to fibre undulation and orientation of undulated fibres aligned a minimum of two axes. To assess the elastic properties of woven composites, several micromechanical models have been presented. Here are a few of the critical articles in this field.

Ishikawa and Chou [1] introduced the undulation, mosaic, and bridging models, which can be used as a basic model for 2D woven fabric composites. Yang et al. [2] proposed a fibre inclination model (i.e., fibre undulation model) in which actual elastic parameters were found utilising a traditional lamination theory-based iso-stress condition approach. Zhang and Harding [3] use the strain energy equivalence theory and the finite element method for the micromechanical analysis of the plain weave composite. A two-dimensional woven fabric (2D-WF) model was developed by Naik and Ganesh [4]. Similar research work was presented by Shembekar and Naik [5] and Ganesh and Naik [6, 7], which was helpful for the prediction of in-plane elastic moduli. The combi-cell model by Vandeurzen et al. [8, 9] can evaluate the shear modulus correctly. Scida et al. [10] developed the MESOTEX model, which used multiple functions to describe fibre geometry and determine the elastic characteristics of woven hybrid composites. Barbero et al. [11] proposed a periodic microstructure model to assess the elasticity of plane wave composites, whereas Barbero et al. [12] reported a comparison between the periodic microstructure model and the finite element model in their research. Donadon et al. [13] established a model for calculating the elastic characteristics of plane weave composites in the warp and weft directions, taking into consideration various undulations and material parameters. They pointed out some inconsistencies in Shembekar and Naik’s model [14] in their research. Barbero [15] and Adumitroaie and Barbero [16] suggested a simple bending restrained model that only required a quarter of a typical unit cell to calculate the thermoelastic properties of a 2D woven fabric composite. In order to analyze the elastic behaviour of 2D plane weave composites, Kowalczyk [17] suggested a typical homogenization technique based on finite elements, which used a periodic-repeated volume element in their modelling techniques. Shokrieh et al. [18] proposed a generalized multiscale micromechanical model to predict the strength and elastic properties of 2D plane weave composites based on the laminate analogy method, in which the entire representative unit cell is divided into sub-domains, and elastic properties are obtained for each domain. Wang et al. [19] used systematic studies to investigate the effects of temperature and strain rate on the stress–strain behaviours of plain and 4-harness satin woven fibre reinforced composites and proposed a constitutive model that accounted for damage progression. Hwang et al. [20] used a genetic algorithm (GA) to improve the elastic modulus of woven fabric composites based on the micromechanics and the geometric modelling presented by Scida et al. [10, 21, 22]

There is very little literature for woven composites when viscoelasticity thermomechanical effects are taken into account. Govindarajan et al. [23] investigated the creep response of woven composites using both experimental and analytical methods. The authors used the correspondence principle and the Maxell-Voigt four-element model to predict the viscoelastic behaviour of the matrix material in their mathematical formulations, which were built on the work of Ishikawa and Chou [24]. Zhu et al. [25] presented and compared a three-dimensional viscoelastic model to estimate time-dependent viscoelastic creep compliance. The relationship between the relaxation property of the matrix material and the time-dependent flexural properties of the fibre bundle was demonstrated in this work. By considering the viscoelastic property of the polyimide matrix, Rupnowski and Kumosa [26] performed a finite element-based stress analysis of eight harness satin/polyimide matrix composites to determine residual stress in a woven composite. Shrotriya and Sottos [27] used dynamic mechanical analysis to investigate the creep responses of woven composites for multilayer printed circuit boards, measuring creep compliance in the warp and fill directions. [21].

Shrotriya and Sottos [28] devised a micromechanical framework for predicting woven composites’ time–temperature dependent material properties for multilayer printed circuit boards. Viscoelastic storage and loss moduli of FR-4 materials utilised in microelectronic devices were determined by Kim [29] using the technique developed by Sottos [30]. The model may predict the relaxation behaviour of the material system in addition to the storage and loss moduli. Greco et al. [31] studied the viscoelastic short term flexural creep response of glass fibre/polypropylene-based woven composites. The linearity of viscoelasticity of the material was examined using Boltzmann's superposition principle and varied stress levels, with the scientists finding that increased temperature resulted in lesser creep compliance. Using the finite element method and volume averaging of three independent repeated volume elements, Naik et al. [32] examined the viscoelastic creep and relaxation behaviour of fibre-based polymer matrix composites. Upadhyaya and Upadhyay [33] devised a 3-D micromechanical model using classical lamination theory and calculated the Prony series coefficients for a variety of samples with varying fibre volume fractions in order to investigate the viscoelastic reactions of woven cloth composites while taking into account the viscoelastic influence of the matrix phase. Jia et al. [34] used micro/mesoscale repetitive unit cells to investigate orthogonal woven composites' low cycle tensile response based on their elastic/viscoelastic properties and characterized the viscoelastic behaviour of resin materials with nonlinear viscoelastic relationship along with switch rules. According to microscopic observations of the weave geometry, a realistic mesoscale finite element model was proposed by Wang et al. [35] to model the matrix's mechanical behaviours; a combined elastoplastic-damage model was proposed. Chen and Aliabadi [36] proposed a novel micromechanical approach for predicting woven kenaf composites' overall nonlinear response and rate-dependent behaviour by employing a viscoelastic model with time-dependent periodic boundary conditions in an explicit way meshfree method to describe the polymer matrix's behaviour. Bhattacharjee et al. [37] proposed an operator-based-approach to explain the viscoelastic characteristics of woven hybrid composites for structural dynamic analysis. Liu et al. [38] developed a data-driven two-step homogenization multiscale modelling approach to predict the effective heat conductivity of two-dimensional (2D) woven composites based on structural genome mechanics. Wu et al. [39] adopted a recursive technique involving laminate theories, mean-field homogenization, Voigt’s rule of mixtures in their study. Bai et al. [40] present a novel micromechanical model based on the minimal total complementary potential energy concept to give a quick and straightforward prediction of the strength of plain weave fabric composites under biaxial strain.

Carbon nanotubes (CNTs) are renowned for their outstanding mechanical characteristics, making them ideal for reinforcing in the polymeric phase [41–46], but relatively little research has been done in the direction of CNTs-reinforced multiscale woven fabric composites. Kim [47] investigated the elastic response of CNTs/carbon fibre/polymer-based multiscale composite materials using numerical and experimental methods. Sadeghi and Pol [48] examined how nanotube content and indenter form affected the tensile and quasi-static punch shear properties of planar woven fibre reinforced composite laminates. Dikshit and Joshi [49] explored the influence of CNTs content on plane woven composite mode-I interlaminar failure, taking into account overall toughening effects from CNTs and fibres. Nam et al. [50] used a multiscale framework to establish the reliability of the Mori–Tanaka based micro-mechanical failure model for radar-absorbing. Swain et al. [51] studied the thermoelastic characteristics of multiscale woven composite materials with radially aligned CNTs and attached with carbon fibre. El Moumen et al. [52] used multiscale homogenization approaches with finite element analysis to predict elastic properties of CNTs based woven fibre reinforced composite and compare them with experimental data. Fantuzzi et al. [53] presented and compared Chamis, Hahn and Halpin–Tsai homogenization technique to predict elastic properties of multiscale woven fabric composites reinforced by carbon nanotubes. Basilisk and Syduzzaman [54] presented an exhaustive review of the different manufacturing processes of CNTs based woven fibre reinforced composites and encapsulated some essential micromechanical techniques for the prediction of thermo-mechanical and electrical properties of such composites.

Guo et al. [55] investigated the effects of multiwalled carbon nanotubes (MWCNT) content on mechanical characteristics of nanocomposite with photosensitive matrix. Wang et al. [56] developed a mathematical model to study the damped response of a nanocomposite plate with stiffeners reinforced with randomly oriented CNT. Huang et al. [57] investigated buckling and dynamics response for graphene-reinforced composites, considering the non-local theory. Li et al. [58] and Al-Furjan et al. [59, 60] conducted similar studies based on the non-local stress–strain gradient theorem. Shen et al. [61] developed the governing differential equations considering modified couple stress theory for dynamic analysis of composite reinforced with graphene nanoplatelets. Researchers derived the governing deferral equation employing the Hamilton principle [62–64] for dynamic analysis of graphene nanoplatelet-reinforced composite. Dai et al. [65] considered a non-polynomial viscoelastic model for the dynamic analysis of open-type composite shells under residual stresses.

Based on a review of the literature, it's evident that further work is needed to develop a complete mathematical framework for analysing the frequency and temperature-dependent viscoelastic characteristics of CNTs-based multiscale 2D woven composites. The study’s primary aim is to propose a unified micromechanical framework to assess the viscoelastic property of a multiscale 2D woven composite reinforced with a randomly oriented carbon nanotube. Apart from this, an attempt was made to address the anisotropic viscoelastic properties arising due to inequal yar thickness. For the sake of completeness, a sincere effort was made to demonstrate the application of the developed micromechanics for the dynamic analysis of structures made of such multiscale composites using the shell finite element method. As a result, the focus of this research is on evaluating the frequency and temperature-dependent viscoelastic characteristics of a two-dimensional woven fabric composite with carbon nanotubes arranged randomly in the polymer phase. First, the Mori–Tanaka micromechanics technique and the viscoelastic weak interphase theory are used to determine the viscoelastic characteristics of nanocomposite. The effect of agglomeration is also taken into account in the micromechanics that have been developed. Following that, in the second mathematical framework, the viscoelastic properties of yarns are formulated using the strength of material method (SOM), and the viscoelastic characteristics of the representative unit cell (RUC) are determined using the unit cell approach (UCM). The elastic properties obtained were compared to those found in the literature for validation. After a two-step validation process, the micromechanics was used to analyse different viscoelastic properties of the proposed composite material system, such as storage moduli (viz. ${E}_{1}{\prime}$, ${E}_{3}{\prime}$, ${G}_{12}{\prime}$, and ${G}_{23}{\prime}$) and In-pane and out of plane loss factors (viz. ${\eta }_{1}, {\eta }_{3}$, ${\eta }_{12}$, and ${\eta }_{23}$). The effect of various parameters such as CNTs content, the interphase condition between CNTs and polymer, fibre content, the geometry of and position of yarn in the representative unit cell on the mentioned viscoelastic properties has been investigated considering an eleven-parameter generalized maxwell model. As the proposed material system shows exceptional material damping characteristics, as one of the main contributions, a comprehensive finite element based dynamic analysis was also performed on shell structures to explain the worthiness of the developed micromechanics. For the purpose of brevity, only linear strain displacement relationships are considered for dynamic analysis.

## Formulation for viscoelastic characteristics of proposed composite

**Fig. 1:**
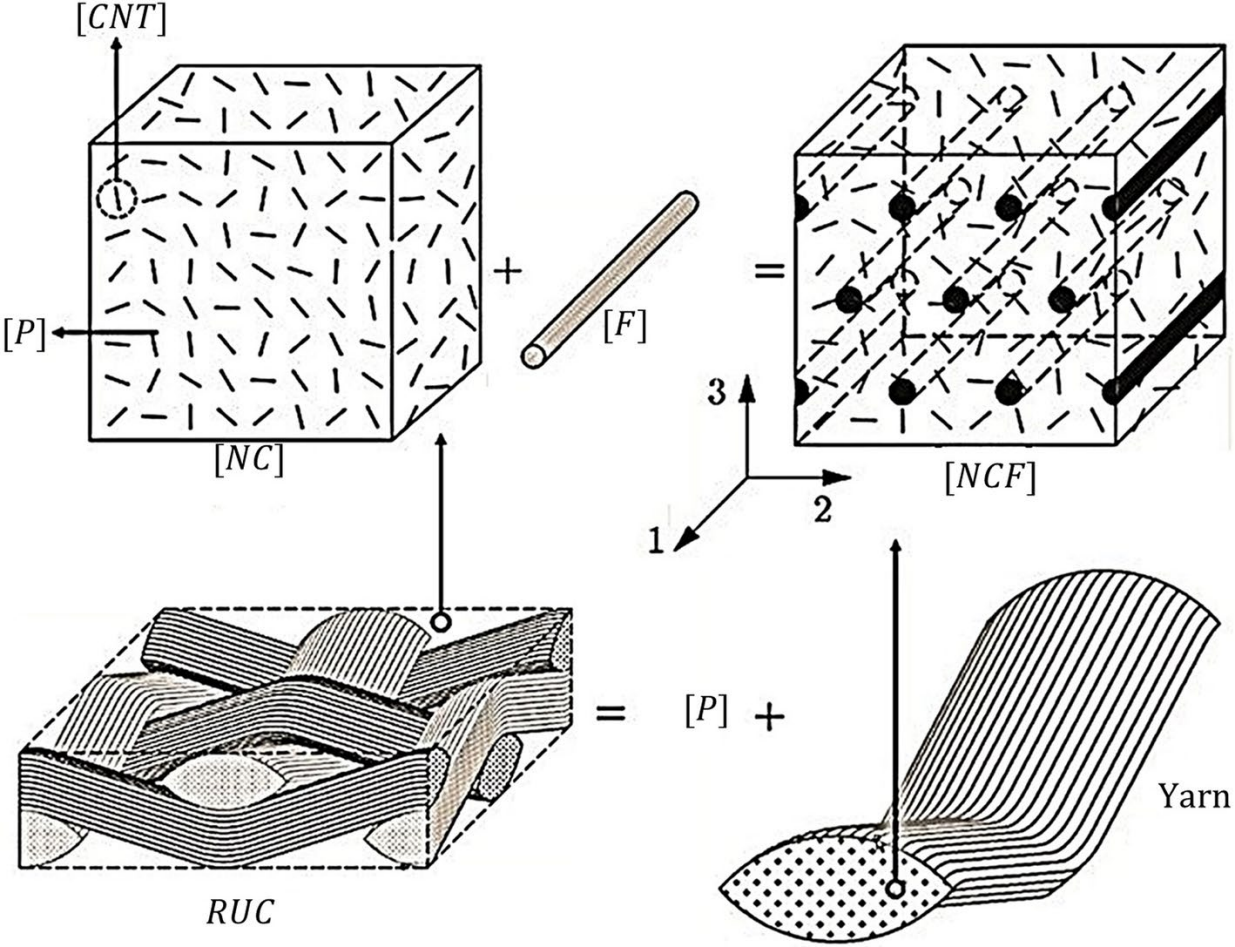
RUC and the constituents of the proposed Hybrid composite

This section describes the constructional features of the proposed nanocomposite hybrid material system and mathematical framework to obtain its viscoelastic properties. Figure 1 depicts the detailed elements of a multiscale material based on carbon fiber/CNTs/polymer and its constituents. It is clear that Carbon nanotubes, carbon fibre, and polymer are the basic building blocks, as seen in Fig. 1. As seen in the figure, the phase $[NC]$ results from randomly oriented CNTs in the polymer phase. When the $[NC]$ phase is coupled with fibre, it produces a specific portion of yarn. When these yarns are combined with $[NC]$, the proposed multiscale composite material is created.

### The correspondence principle and viscoelasticity

The matrix and CNTs are the two primary phases of the nanocomposite, with the matrix being isotropic and the CNTs being transversely isotropic. Among these two constituent phases, the matrix is viscoelastic. Using the correspondence principle, the constitutive relationship for the viscoelastic matrix phase can be represented as follows [66]:

1 $${\sigma }^{P}\left(t\right)={\int }_{0}^{t}{C}^{P}\left(t-\Gamma \right):{\dot{\varepsilon }}^{P}\left(\Gamma \right)d\Gamma$$

where ${\sigma }^{P}$, ${\dot{\varepsilon }}^{P}$ and ${C}^{P}$ indicate the matrix phase’s stress, strain rate, and stiffness, respectively.

In this case, the half-sided Fourier transformation is applied, which is given by

2 $$\underline{f} \left( \omega \right) = \mathop \smallint \limits_{0}^{\infty } f\left( t \right)e^{ - i\omega t} dt$$

As a result, the frequency domain form of the Eq. (1) is as follows:

3 $${\underset{\_}{\sigma }}^{P}\left(\omega \right)=i\omega {\underset{\_}{\underline{C}}}^{P}\left(\omega \right):{\underset{\_}{\varepsilon }}^{P}\left(\omega \right)$$

The Eq. (3) can alternatively be written in the following way:

4 $${\underset{\_}{\sigma }}^{P}\left(\omega \right)={\underline{C}}^{P*}\left(\omega \right):{\underset{\_}{\varepsilon }}^{P}\left(\omega \right)$$

The underscore symbol $\underset{\_}{{\phantom{a}}}$ indicates that the parameter is a frequency function rather than a time function, while the star symbol * indicates that the parameter is a complex number. These symbols are not used for brevity because the mechanics hold true in the time and frequency domain.Since the CNT is completely elastic, Hook’s law can be represented by fourth-order stiffness tensors ${C}^{CNT}$, which can be related to stress and strain as follows.

5 $${\sigma }^{CNT}={C}^{CNT}:{\varepsilon }^{CNT}$$

### Mori–Tanaka micromechanics

**Fig. 2:**
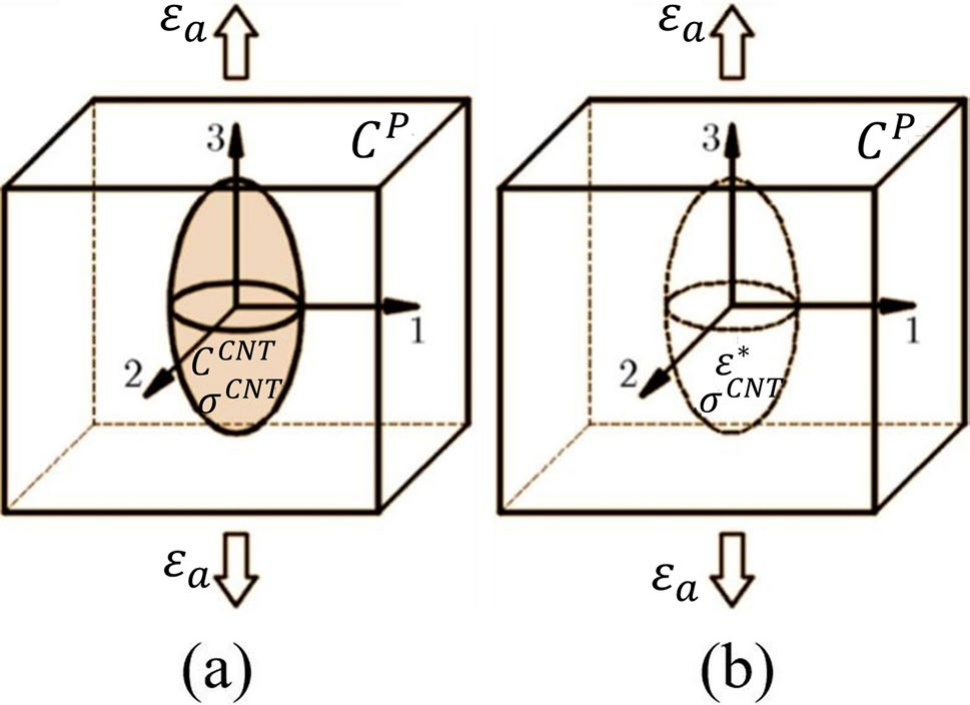
**a** RVE with inclusion and **b** comparison material without inclusion for Eshelby’s Equivalent problem

Nanocomposite stiffness tensor (i.e., ${C}^{NC}$) is evaluated using the MT approach and approximated to be ellipsoidal in the form [67–79]. Figure 2 shows Eshelby's equivalent inclusion problem, with Fig. 2a illustrating the nanocomposite and Fig. 2b illustrating the matrix. In Fig. 2b, the black dotted line represents the volume that the inclusion could occupy.

Average developed stresses in both the domain due to the same average strain (${\varepsilon }_{a}$) caused with a displacement at the boundary

6 $$\begin{array}{c}{\tilde{\sigma }}^{NC}={C}^{NC}:{\varepsilon }_{a}\\ {\tilde{\sigma }}^{P}={C}^{P}:{\varepsilon }_{a}\end{array}$$

In the configurations of ‘a’ and ‘b’ the average state of stresses are ${\tilde{\sigma }}^{NC}$ and ${\tilde{\sigma }}^{P}$ respectively. An average parameter is represented by the bars or over-score. The existence of inclusion causes the strain field in the matrix to become non-uniform, as demonstrated in Fig. 2a. The average strain state in the matrix can be represented by the equation below.

7 $${\overline{\varepsilon }}^{P}={\varepsilon }_{a}+{\overline{\varepsilon }}^{P pt}$$

As a result, the inclusion phase can go through a similar change, and the average state of strain in the inclusion can be represented by

8 $${\overline{\varepsilon }}^{CNT}={\overline{\varepsilon }}^{P}+{\varepsilon }^{CNT pt}$$

The abbreviation $pt$ stands for perturbation. The relationship between the inhomogeneous inclusion in Fig. 2a and the homogeneous inclusion problem in Fig. 2b may be demonstrated by correlating the matrix and inclusion properties as follows, as stated by Eshelby [80]

9 $${\tilde{\sigma }}^{CNT}={C}^{CNT}:{\overline{\varepsilon }}^{CNT}={C}^{P}:\left({\overline{\varepsilon }}^{CNT}-{\varepsilon }^{*}\right)$$

where, ${\varepsilon }^{*}$ denotes the inclusion's Eigen-strain, which is related to the inclusion perturbation strain by the equation

10 $${\varepsilon }^{CNT pt}=S:{\varepsilon }^{*}$$

In Eq. (10) the Eshelby's tensor is denoted by $S$, and related words are included in Appendix A. The Eigen-strain in Eq. (9) may be readily derived as

11 $${\varepsilon }^{*}=-{S}^{P}:\left({C}^{CNT}-{C}^{P}\right):{\overline{\varepsilon }}^{CNT}$$

In Eq. (11) the compliance tensor for polymer materials is ${S}^{P}$. By substituting Eq. (11) in Eq. (8) and using Eq. (10) the average state of strain of inclusion can be related to the average state of strain of matrix as

12 $${\overline{\varepsilon }}^{CNT}={\left[I+S:{S}^{P}:\left({C}^{CNT}-{C}^{P}\right)\right]}^{-1}:{\overline{\varepsilon }}^{P}$$

The tensor that relates average strain in the inclusion to average strain in the matrix in Eq. (12) is known as the dilute strain concentration tensor and can be written as

13 $${A}^{\text{dil}}={\left[I+S:{S}^{P}:\left({C}^{CNT}-{C}^{P}\right)\right]}^{-1}$$

$I$ represents the fourth-order identity tensor in the previous Eqs. (12) and (13). Equation (12) may be written as

14 $${\overline{\varepsilon }}^{CNT}={A}^{\text{dil}}:{\overline{\varepsilon }}^{P}$$

The average strain ${\varepsilon }_{a}$ contributes to the inclusion and matrix’s average strain.

15 $${v}_{NC}^{P}{\overline{\varepsilon }}^{P}+{v}_{NC}^{CNT}{\overline{\varepsilon }}^{CNT}={\varepsilon }_{a}$$

In Eq. (15) the volume fractions of matrix and carbon nano tubes in the nano composite phase are ${v}_{NC}^{P}$ and, ${v}_{NC}^{CNT}$ respectively. By substituting Eq. (12) into Eq. (15), will give

16 $${\overline{\varepsilon }}^{P}={\left[{v}_{NC}^{P}I+{v}_{NC}^{CNT}{A}^{\text{dil}}\right]}^{-1}:{\varepsilon }_{a}$$

It is worth noting that the applied average strain and the matrix’s average strain are now linked, and the Equation may express the strain concentration tensor. (17)

17 $${A}_{0}={\left[{v}_{NC}^{P}I+{v}_{NC}^{CNT}{A}^{\text{dil}}\right]}^{-1}$$

As a result, Eq. (16) may be written as

18 $${\overline{\varepsilon }}^{P}={A}_{0}:{\varepsilon }_{a}$$

After putting Eq. (18) in Eq. (14), the average strain tensor in the inclusion can be written in terms of applied average strains and average strains in the inclusion becomes

19 $${\overline{\varepsilon }}^{CNT}={A}^{\text{dil}}:{A}_{0}:{\varepsilon }_{a}={A}^{\text{ndil}}:{\varepsilon }_{a}$$

where, the non-dilute strain concentration tensor in Eq. (19) is denoted by, ${A}^{\text{ndil}}$. Similar to Eq. (15), in the case of an aligned inclusion, the average stress tensor of nanocomposite may be obtained as

20 $${v}_{NC}^{P}{\tilde{\sigma }}^{P}+{v}_{NC}^{CNT}{\tilde{\sigma }}^{CNT}=\tilde{\sigma }={C}^{NC}:{\varepsilon }_{a}$$

The effective stiffness tensor of the nanocomposite may be obtained by rearranging Eq. (20) with the information that, ${\tilde{\sigma }}^{P}={C}^{P}:{\overline{\varepsilon }}^{P}$ and $\overline{\sigma }^{CNT} = C^{CNT} :\overline{\varepsilon }^{CNT}$.

21 $${C}^{NC}=\left({v}_{NC}^{P}{C}^{P}+{v}_{NC}^{CNT}{C}^{CNT}:{A}^{\text{dil}}\right):{\left({v}_{NC}^{P}I+{v}_{NC}^{CNT}{A}^{\text{dil}}\right)}^{-1}$$

The strain and stress of a CNTs or inclusion may be averaged in all conceivable directions for randomly oriented CNTs, and so Eq. (14) can be expressed as

22 $$\begin{array}{c}\langle {\overline{\varepsilon }}^{CNT}\rangle =\langle {A}^{\text{dil}}:{\overline{\varepsilon }}^{P}\rangle =\langle {A}^{\text{dil}}\rangle :{\overline{\varepsilon }}^{P}\\ \langle {\tilde{\sigma }}^{CNT}\rangle =\langle {C}^{CNT}:{A}^{\text{dil}}\rangle :{\overline{\varepsilon }}^{P}\end{array}$$

Hence for randomly oriented CNTs, the Eq. (21) can be modified as

23 $${C}^{NC}=\left({v}_{NC}^{P}{C}^{P}+{v}_{NC}^{CNT}\langle {C}^{CNT}{A}^{\text{dil}}\rangle \right):{\left({v}_{NC}^{P}I+{v}_{NC}^{CNT}\langle {A}^{\text{dil}}\rangle \right)}^{-1}$$

The details about the bracket $\langle \rangle$ is given in Sect. 2.3.

### Weak interface theory

To represent the WI, Qu [81] and Esteva and Spanos [76] used a linear spring layer with a negligible thickness, where traction was assumed to be continuous, and displacement was assumed to be discontinuous at the interfaces. The following equation may be used to represent both continuous traction and displacement jump.

24 $$\begin{array}{c}\Delta {\sigma }_{ij}{n}_{j}\equiv \, \, [{\sigma }_{ij}({{S}_{I}}^{+})-{\sigma }_{ij}({{S}_{I}}^{-})]{n}_{j}=0\\ \Delta {u}_{i}\equiv \, \, [{u}_{i}({{S}_{I}}^{+})-{u}_{i}({{S}_{I}}^{-})] \, \, ={\eta }_{ij}{\sigma }_{jk}{n}_{k}\end{array}$$

The parameters ${S}_{I}$ in the Eq. (24) indicate the interface and $n$ is a unit vector that is normal and outward from it. ${u}_{i}({{S}_{I}}^{+})$ and ${u}_{i}({{S}_{I}}^{-})$ are the displacement amounts while approaching the inclusion from the inside and outside, respectively. The second-order (${\eta }_{ij}$) accounts for the spring layer's compliance and is expressed as

25 $${\eta }_{ij}=\alpha {\delta }_{ij}+(\beta -\alpha ){n}_{i}{n}_{j}$$

**Fig. 3:**
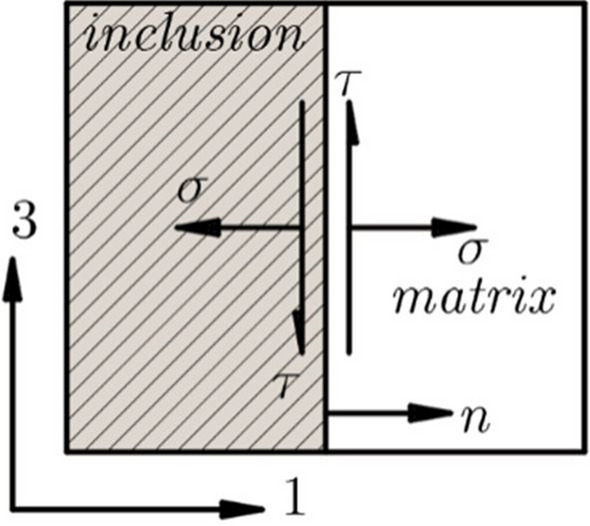
Illustration of for WI

The cut section of CNTs inclusion in the polymer matrix is shown in Fig. 3, where both the normal and shear stresses are produced due to loading. Normal stress is generated in direction 1, whereas shear stress is induced in direction 3 at the thickness less interface. For the sake of simplicity in this problem, all stress components in the other direction are ignored. Hence all terms i.e., ${\eta }_{ij}$ ( $i,j=2$) are ignored.

The indicial notations can be used to write expansions of the second part of the Eq. (24) for the nanocomposite section (shown in Fig. 3)

.

26 $$\begin{array}{c}\Delta {u}_{1}={\eta }_{11}{\sigma }_{11}{n}_{1}+{\eta }_{11}{\sigma }_{13}{n}_{3}+{\eta }_{13}{\sigma }_{31}{n}_{1}+{\eta }_{13}{\sigma }_{33}{n}_{3}\\ \Delta {u}_{3}={\eta }_{31}{\sigma }_{11}{n}_{1}+{\eta }_{31}{\sigma }_{13}{n}_{3}+{\eta }_{33}{\sigma }_{31}{n}_{1}+{\eta }_{33}{\sigma }_{33}{n}_{3}\end{array}$$

In Eq. (26) ${n}_{3}=0$ and ${n}_{1}=1$. when the inclusion is oriented along direction ‘3’ (as illustrated in Fig. 3). As a result, Eq. (26) is reduced to

27 $$\begin{array}{c}\Delta {u}_{1}={\eta }_{11}{\sigma }_{11}{n}_{1}+{\eta }_{13}{\sigma }_{31}{n}_{1}\\ \Delta {u}_{3}={\eta }_{31}{\sigma }_{11}{n}_{1}+{\eta }_{33}{\sigma }_{31}{n}_{1}\end{array}$$

The terms, ${\eta }_{11}$,${\eta }_{13}$,${\eta }_{31}$ and ${\eta }_{33}$. may be derived further from Eq. (25) as follows

28 $$\begin{array}{c}{\eta }_{11}=\alpha {\delta }_{11}+(\beta -\alpha ){n}_{1}{n}_{1}=\beta \\ {\eta }_{13}=\alpha {\delta }_{13}+(\beta -\alpha ){n}_{1}{n}_{3}=0\\ {\eta }_{31}=\alpha {\delta }_{31}+(\beta -\alpha ){n}_{3}{n}_{1}=0\\ {\eta }_{33}=\alpha {\delta }_{33}+(\beta -\alpha ){n}_{3}{n}_{3}=\alpha \end{array}$$

Putting Eq. (28) in Eq. (27), which produces

29 $$\begin{array}{c}\Delta {u}_{1}=\beta \sigma \\ \Delta {u}_{3}=\alpha \tau \end{array}$$

The Eq. (29) may be used to calculate the tangential and normal compliances (such as $\alpha$ and $\beta$). The Eshelby's tensor may be calculated using WI theory [76, 81] as

30 $${\overline{S} }_{ijkl}={S}_{ijkl}+\left({I}_{ijpq}-{S}_{ijpq}\right){H}_{pqrs}{C}_{rsmn}^{P}\left({I}_{mnkl}-{S}_{mnkl}\right)$$

where $H$ is given by

31 $${H}_{ijkl}=\alpha {P}_{ijkl}+(\beta -\alpha ){Q}_{ijkl}$$

Appendix B contains a list of other unknown terms in the Eq. (31). As a result, Eq. (21) is rewritten to become

32 $$\begin{array}{c}{C}^{NC}=\left({v}_{NC}^{P}{C}^{P}+{v}_{NC}^{CNT}{C}^{CNT}:{\overline{A} }^{\text{dil}}\right):\\ {\left[{v}_{NC}^{P}I+{v}_{NC}^{CNT}\left({\overline{A} }^{\text{dil}}+H:{C}^{CNT}:{\overline{A} }^{\text{dil}}\right)\right]}^{-1}\end{array}$$

were,

33 $${\overline{A} }^{\text{dil}}={\left[I+\overline{S }:{S}^{P}:\left({C}^{CNT}-{C}^{P}\right)\right]}^{-1}$$

At this point Eq. (32) gives the property of nanocomposite or modified matrix which is transversely isotropic in nature depending on the orientation of inclusion. For modified matrix or the nanocomposite phase where the inclusions with weak interphase are randomly oriented, the effective property can be obtained by averaging the property as done in Eq. (23). So the Eq. (32) for randomly oriented inclusion may be written as

34 $$\begin{array}{c}{C}^{NC}=\left({v}_{NC}^{P}{C}^{P}+{v}_{NC}^{CNT}\langle {C}^{CNT}:{\overline{A} }^{\text{dil}}\rangle \right)\\ :{\left({v}_{NC}^{P}I+{v}_{NC}^{CNT}\langle {\overline{A} }^{\text{dil}}\rangle +{v}_{NC}^{CNT}\langle H:{C}^{CNT}:{\overline{A} }^{\text{dil}}\rangle \right)}^{-1}\end{array}$$

**Fig. 4:**
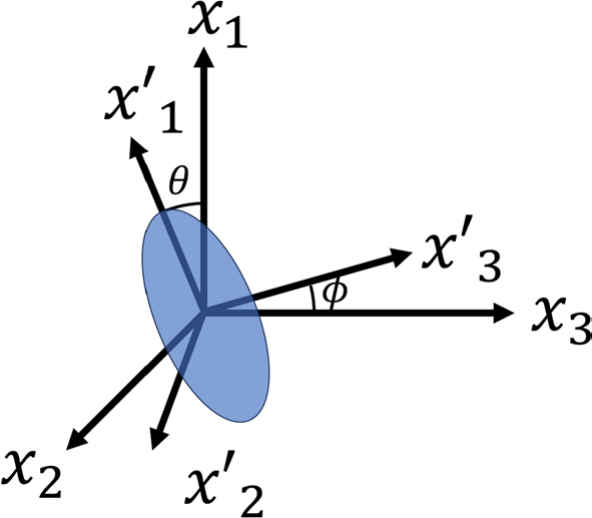
Inclusion in local and global coordinate system

Both in Eqs. (23) and (34) the terms within this bracket $\langle \rangle$ indicate that it has been averaged over the volume. To predict the behaviour of a system characterized by a three-dimensional random fiber orientation, it is necessary to establish a correspondence between the local coordinates of particles and the global coordinates of composites, as seen in Fig. 4. The primed coordinate system represents the local axis of a spheroid inclusion, whereas the unprimed coordinate system represents the global. If the axis of the inclusion is along the ${x}_{1}{\prime}$. The co-ordinate transformation from the local ${\varvec{x}}\boldsymbol{^{\prime}}$ to global ${\varvec{x}}$ can be represented by $\left\{{x}_{i}{\prime}\right\}=\left[{Q}_{ij}\right]\left\{{x}_{j}\right\}$. Where $\left[Q\right]$ is given as follows [82].

35 $$\left[\begin{array}{ccc}\mathit{cos}\theta & \mathit{sin}\theta \mathit{cos}\phi & \mathit{sin}\theta \mathit{sin}\phi \\ -\mathit{sin}\theta & \mathit{cos}\theta \mathit{cos}\phi & \mathit{cos}\theta \mathit{sin}\phi \\ 0& -\mathit{sin}\phi & \mathit{cos}\phi \end{array}\right]$$

As per Fig. 4, the $\theta$ can vary from 0 to $\pi /2$ where $\phi$ can be varied from 0 to $2\pi$ for any possible orientation in the global coordinate. Therefore, the determination of any material constants $\langle {L}_{ijkl}\rangle$ for a composite with 3D randomly oriented inclusions may be achieved using equation Eq. (36).

36 $$\langle {L}_{ijkl}\rangle = \frac{1}{2\pi }\underset{0}{\overset{2\pi }{\int }}{\int }_{0}^{\frac{\pi }{2}}{Q}_{mi}{Q}_{nj}{L}_{mnpq}{Q}_{pk}{Q}_{ql}{\text{sin}}\theta d\theta d\phi$$

### Agglomeration effects of CNTs

**Fig. 5:**
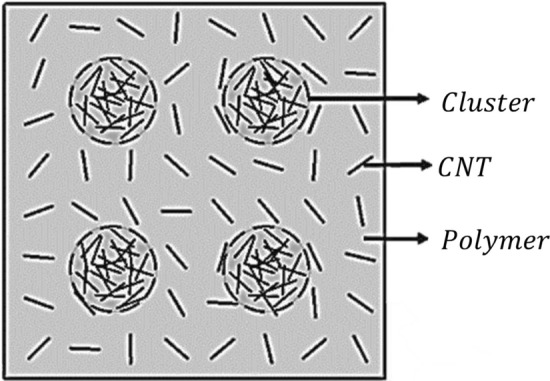
RVE showing CNT, clusters and polymer phases

When the fraction of CNTs in the polymer phase gets large, there is always the possibility of CNTs clubbing to form clusters in the polymer. This is referred to as CNTs aggregation/ agglomeration. Figure 5 shows the formed cluster in the nanocomposite phase.

37 $${V}_{NC}^{CNT}={V}_{cluster}^{CNT}+{V}_{P}^{CNT}$$

The cluster is a spherical inclusion with randomly arranged transversely isotropic CNTs, resulting cluster’s property being isotropic. The resultant phase must be isotropic since the isotropic clusters are present in an isotropic phase (specifically *NC*). If ${V}_{NC}^{CNT}$ represent the total volume of total CNTs, and it may be separated into two components [75]. Where, ${V}_{Cluster}^{CNT}$ represents the volume of CNTs trapped inside the cluster, and, ${V}_{P}^{CNT}$ represents the remaining CNTs in the polymer phase outside of the cluster. The following are the agglomeration constants [75] defined as

38 $$\xi = \frac{{V^{{cluster}} }}{{V^{{NC}} }};\quad \zeta {\text{ = }}\frac{{{\text{V}}_{{{\text{cluster}}}}^{{{\text{CNT}}}} }}{{{\text{V}}_{{{\text{NC}}}}^{{{\text{CNT}}}} }}$$

As a result, $\xi$ denotes the volume fraction of the spherical cluster relative to the RVE, and $\zeta$ represents the volume of CNTs trapped in the spherical cluster as a percentage of total CNTs in the RVE. As per Eq. (38), CNTs are said to be uniformly dispersed in the matrix when $\xi =1$ CNTs are uniformly dispersed in the matrix and the equation also implies that lower the value of $\xi$, the more severe will be the degree of CNTs aggregation. When $\zeta =1$, all the CNTs are considered to be concentrated in the inclusion, but as $\zeta$ decreases, the concentration of CNTs in the cluster declines. CNTs, on the other hand, are are evenly dispersed throughout the matrix when $\xi =\zeta$. From Eqs. (37) and (38) can be solved for the volume fraction of CNTs trapped inside the cluster with respect to the spherical cluster and can be represented as

39 $${v}_{cluster}^{CNT}=\frac{\zeta }{\xi }{v}_{NC}^{CNT}$$

As a result, the elastic property of the spherical cluster may be determined using the equation below.

40 $$\begin{array}{c}{\left.{C}^{NC}\right|}_{in}=\left((1-{v}_{cluster}^{CNT}){C}^{P}+{v}_{cluster}^{CNT}\langle {C}^{CNT}:{\overline{A} }^{\text{dil}}\rangle \right):\\ {\left((1-{v}_{cluster}^{CNT})I+{v}_{cluster}^{CNT}\langle {\overline{A} }^{\text{dil}}\rangle +{v}_{cluster}^{CNT}\langle H:{C}^{CNT}:{\overline{A} }^{\text{dil}}\rangle \right)}^{-1}\end{array}$$

The volume fraction of CNTs in the polymer phase but outside the cluster may be calculated to be

41 $${v}_{P}^{CNT}=\left(\frac{1-\zeta }{1-\xi }\right){v}_{NC}^{CNT}$$

Based on Eq. (41), the elastic property outside of the spherical cluster may be computed as

42 $$\begin{array}{c}{\left.{C}^{NC}\right|}_{out}=\left((1-{v}_{p}^{CNT}){C}^{P}+{v}_{p}^{CNT}\langle {C}^{CNT}:{\overline{A} }^{\text{dil}}\rangle \right):\\ {\left((1-{v}_{p}^{CNT})I+{v}_{p}^{CNT}\langle {\overline{A} }^{\text{dil}}\rangle +{v}_{p}^{CNT}\langle H:{C}^{CNT}:{\overline{A} }^{\text{dil}}\rangle \right)}^{-1}\end{array}$$

Finally, the effective elastic property of the *NC* phase may be determined, which takes into account the weak interphase and agglomeration as [66, 83, 84]

43 $$\begin{array}{c}{C}^{NC}=\left((1-\xi ){\left.{C}^{NC}\right|}_{out}+\xi \langle {\left.{C}^{NC}\right|}_{in}:{\stackrel{-}{\overline{A}} }^{\text{dil}}\rangle \right):\\ {\left((1-\xi )I+\xi \langle {\stackrel{-}{\overline{A}} }^{\text{dil}}\rangle +\xi \langle H{\left.:{C}^{NC}\right|}_{in}:{\stackrel{-}{\overline{A}} }^{\text{dil}}\rangle \right)}^{-1}\end{array}$$

and

44 $${\stackrel{-}{\overline{A}} }^{\text{dil}}={\left[I+\overline{S }{\left.:{S}^{NC}\right|}_{out}:\left({\left.{C}^{NC}\right|}_{in}-{\left.{C}^{NC}\right|}_{out}\right)\right]}^{-1}$$

### Correspondence principle for the weak interface

The compliances (${\eta }_{ij}$) in Eq. (25) for a viscoelastic material are time-dependent, and this can be achieved by representing $\alpha$ and $\beta$ as a function of time. As a result, Eq. (25) may be written as

45 $${\eta }_{ij}(t)=\alpha (t){\delta }_{ij}+(\beta (t)-\alpha (t)){n}_{i}{n}_{j}$$

As a result, the displacement jump becomes time-dependent and can be represented as

46 $$\Delta {u}_{i}(t)=\underset{-\infty }{\overset{t}{\int }}{\eta }_{ij}(t-\Gamma )\frac{\partial {\sigma }_{jk}(\Gamma )}{\partial \Gamma }d\Gamma .\Delta {n}_{k}$$

Invoking Eq. (2), the correspondence principle may be used to transform Eq. (46) to the frequency domain, and it can be expressed as

47 $$\Delta {u}_{i}(\omega )={\int }_{0}^{\infty }\left[{\int }_{-\infty }^{t}{\eta }_{ij}(t-\Gamma )\frac{\partial {\sigma }_{jk}(\Gamma )}{\partial \Gamma }d\Gamma \right]{e}^{-i\omega t}dt.\Delta {n}_{k}$$

Which results

48 $$\begin{array}{c}\Delta {u}_{1}(\omega )={\int }_{0}^{\infty }\left[{\int }_{-\infty }^{t}\beta (t-\Gamma )\frac{\partial \sigma (\Gamma )}{\partial \Gamma }d\Gamma \right]{e}^{-i\omega t}dt\cdot\Delta {n}_{3}\\ \Delta {u}_{3}(\omega )={\int }_{0}^{\infty }\left[{\int }_{-\infty }^{t}\alpha (t-\Gamma )\frac{\partial \tau (\Gamma )}{\partial \Gamma }d\Gamma \right]{e}^{-i\omega t}dt\cdot\Delta {n}_{1}\end{array}$$

The viscoelastic compliance of and is frequency dependent $\alpha$ and $\beta$ may be expressed as

49 $$\begin{gathered} \alpha \left( \omega \right) = \alpha ^{\prime } \left( \omega \right) - i\alpha ^{{\prime \prime }} \left( \omega \right) \hfill \\ \beta \left( \omega \right) = \beta \prime \left( \omega \right) - i\beta ^{{\prime \prime }} \left( \omega \right) \hfill \\ \end{gathered}$$

For CNTs, $\beta$ can be assumed to be zero [66, 76, 83–85], and the compliance $\alpha$ can be represented in terms of a dimensionless parameter ${\alpha }_{0}$, diameter of CNTs and viscoelastic shear modulus $G\left(\omega \right)$ of polymer phase as follows

50 $$\alpha \left( \omega \right) = \frac{{\alpha _{0} d_{{cnt}} }}{{\left| {G\left( \omega \right)} \right|}}\left\{ {1 - i\frac{{\alpha ^{{\prime \prime }} }}{{\alpha ^{\prime } }}} \right\}$$

Here, ${d}_{cnt}$ is the diameter of CNTs and $\alpha_{0}$ implies the magnitude of ${\alpha }{\prime}$ whereas the ratio ${\alpha }^{^{\prime\prime} }/{\alpha }{\prime}$ represents interfacial loss factor.

### Elastic properties of individual yarn

SOM may be used to determine the effective viscoelastic properties of $NCF$ phase which is formed by combining $NC$ phase and along with $F$*.* As shown in Fig. 1, the axis of transverse isotropy along axis '1' while considering $NCF$ phase. Considering the iso-field condition and the rules of mixture, the state of stresses and strains for the individual constituents’ $NC$ and $F$ phases are related to the state of stress and strain of $NCF$ phase*.*

51 $$\begin{array}{c}{\varepsilon }_{1}^{NCF}={\varepsilon }_{1}^{NC}={\varepsilon }_{1}^{F}\\ \left\{\begin{array}{c}{\sigma }_{2}^{NCF}\\ {\sigma }_{3}^{NCF}\\ {\sigma }_{23}^{NCF}\\ {\sigma }_{13}^{NCF}\\ {\sigma }_{12}^{NCF}\end{array}\right\}=\left\{\begin{array}{c}{\sigma }_{2}^{NC}\\ {\sigma }_{3}^{NC}\\ {\sigma }_{23}^{NCF}\\ {\sigma }_{13}^{NC}\\ {\sigma }_{12}^{NC}\end{array}\right\}=\left\{\begin{array}{c}{\sigma }_{2}^{F}\\ {\sigma }_{3}^{F}\\ {\sigma }_{23}^{F}\\ {\sigma }_{13}^{F}\\ {\sigma }_{12}^{F}\end{array}\right\}\\ {\sigma }_{1}^{NCF}={v}_{NCF}^{NC}{\sigma }_{1}^{NC}+{v}_{NCF}^{F}{\sigma }_{1}^{F}\\ \left\{\begin{array}{c}{\varepsilon }_{2}^{NCF}\\ {\varepsilon }_{3}^{NCF}\\ {\varepsilon }_{23}^{NCF}\\ {\varepsilon }_{13}^{NCF}\\ {\varepsilon }_{12}^{NCF}\end{array}\right\}={v}_{NCF}^{NC}\left\{\begin{array}{c}{\varepsilon }_{2}^{NC}\\ {\varepsilon }_{3}^{NC}\\ {\varepsilon }_{23}^{NC}\\ {\varepsilon }_{13}^{NC}\\ {\varepsilon }_{12}^{NC}\end{array}\right\}+{v}_{NCF}^{F}\left\{\begin{array}{c}{\varepsilon }_{2}^{F}\\ {\varepsilon }_{3}^{F}\\ {\varepsilon }_{23}^{F}\\ {\varepsilon }_{13}^{F}\\ {\varepsilon }_{12}^{F}\end{array}\right\}\end{array}$$

where ${v}_{NCF}^{NC}$ and ${v}_{NCF}^{F}$ are the volume fraction of $NC$ and carbon fibre ($F$) with respect to $NCF$ which is related by ${v}_{NCF}^{NC}+{v}_{NCF}^{F}=1$*.*The constitutive relation of individual constituents in $NCF$ can be written as

52 $$\left\{ {\sigma ^{k} } \right\} = \left[ {C^{k} } \right]\left\{ {\varepsilon ^{k} } \right\};\quad k = NC,F\,and\,NCF$$

The Eqs. (51) and (52) together give the constitutive relation for $PMNCF$ as follows

53 $$\left[{C}^{NCF}\right]=\left[{C}_{1}\right]{\left[{V}_{1}\right]}^{-1}+\left[{C}_{2}\right]{\left[{V}_{2}\right]}^{-1}$$

The individual terms used in Eq. (53) such as $\left[{C}_{1}\right],$ $\left[{V}_{1}\right],$ $\left[{C}_{2}\right]$ and $\left[{V}_{2}\right]$ are defined in the Appendix C.

### Elastic properties of the representative unit cell (${\varvec{R}}{\varvec{U}}{\varvec{C}}$)

**Fig. 6:**
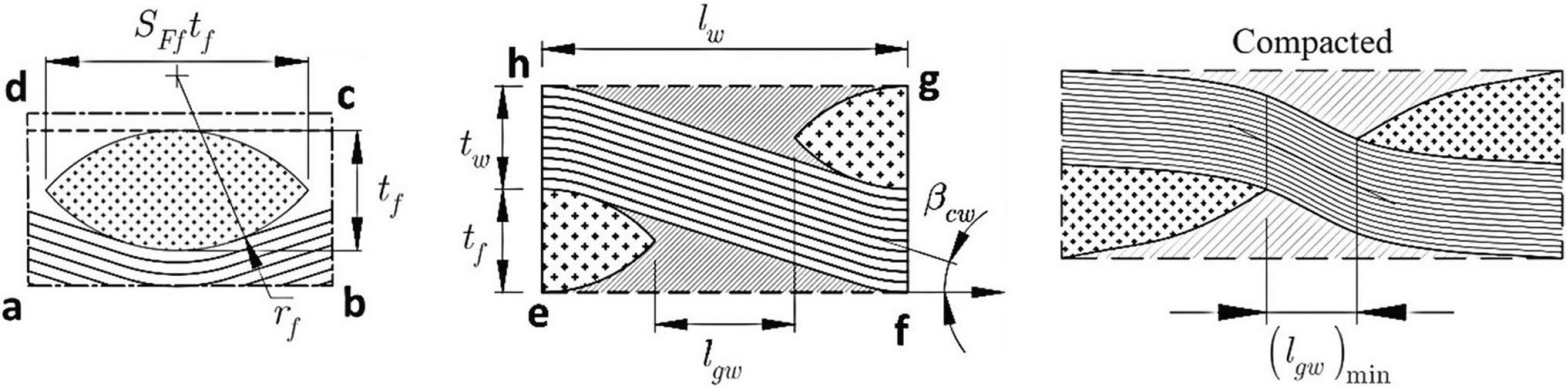
Sectional view of *RUC*

Figure 6 shows the detailed constructional feature of the $RUC$. As can be seen from Fig. 6

the fill yarn, warp yarn, and the polymer are the $RUC$'s primary constituents. The yarns are lenticular in cross-section with the radius of curvature, thickness, and breadth given by ${r}_{k}$, ${t}_{k}$ and ${S}_{Fk}{t}_{k}$ respectively, as shown in section ‘a-b-c-d-a’. It may be deduced from section ‘e-f-g-h-e’ that the yarn-to-yarn distance is ${l}_{k}$ and the gap between two yarns is ${l}_{gk}$ and ${\left({l}_{gk}\right)}_{{\text{min}}}$ is the smallest possible gap between two yarns. The crimp angle of the yarn is represented by ${\beta }_{ck}$ where, subscript *k* represents either the warp or fill ($k=w,f$). The radius, inner angle, and area of a lenticular wrap yarn may be represented based on the geometry of lenticular sectional view as

54 $$\begin{gathered} r_{w} = \frac{{t_{w} \left( {1 + S_{{Fw}}^{2} } \right)}}{4} \hfill \\ \alpha _{w} = 2\arcsin \left( {\frac{{2S_{{Fw}} }}{{1 + S_{{Fw}}^{2} }}} \right) \hfill \\ A_{w} = r_{w}^{2} \left( {\alpha _{w} - \sin \alpha _{w} } \right) \hfill \\ \end{gathered}$$

The yarn to yarn distance is clearly shown in Fig. 6 as

55 $${L}_{w}={S}_{Ff}{t}_{f}+{L}_{gw}$$

It is also worth noting that, as shown in Fig. 6, the specified gap length for a tightly packed/ compressed composite laminate cannot be zero. For a tightly packed/ compressed composite laminate, the shortest gap length is

56 $${\left({L}_{gw}\right)}_{{\text{min}}}=\left(2{r}_{f}+{t}_{w}\right){\text{sin}}{\beta }_{w}-{S}_{Ff}{t}_{f}$$

57 $${\text{where}}, {\left.{\beta }_{w}\right|}_{{\left({L}_{gw}\right)}_{{\text{min}}}}={\text{arccos}}\left(\frac{2{r}_{f}+{t}_{w}-{t}_{f}}{2{r}_{f}+{t}_{w}}\right)$$

The yarn crimp angle for wrap yarn (${\beta }_{cw}$) may be calculated as

58 $$\begin{gathered} \beta _{{cw}} = \arcsin \left( {\frac{{2r_{f} + t_{w} }}{{\sqrt {L_{w}^{2} + \left( {2r_{f} + t_{w} - t_{f} } \right)^{2} } }}} \right) - \beta _{{ow}} \hfill \\ where,{\mkern 1mu} \beta _{{ow}} = \arctan \left( {\frac{{2r_{f} + t_{w} - t_{f} }}{{L_{w} }}} \right) \hfill \\ \end{gathered}$$

The length partition of the crimp part with respect to the total length of wrap yarn can be expressed as

59 $${\mu }_{cw}=\frac{\left(2{r}_{f}+{t}_{w}\right){\text{sin}}\left({\beta }_{cw}\right)}{{L}_{w}}$$

where the straight portion length of the wrap yarn is expressed as

60 $${L}_{sw}=\sqrt{{L}_{w}^{2}+{t}_{f}^{2}-2{t}_{f}\left(2{r}_{f}+{t}_{w}\right)}$$

As a result, the volume of the wrap yarn is calculated using the lengths of the curved, the straight portions, and the area of the wrap yarn, which may be expressed as

61 $${V}_{yw}=4{A}_{w}\left\{\left(2{r}_{f}+{t}_{w}\right){\beta }_{cw}+{L}_{sw}\right\}$$

Similarly, by changing the subscript from ‘$w$’ to ‘$f$’ in Eqs. (54)–(61), one can get different fill yarn parameters. Given the volume of the RUC ${V}_{RUC}=4\left({t}_{w}+{t}_{f}\right){L}_{w}{L}_{f}$ the actual volume fraction of $NCF$ with respect to the RUC can be calculated using the following expression.

62 $${v}_{RUC}^{NCF}=pf\times \frac{{V}_{yf}+{V}_{yw}}{{V}_{RUC}}$$

where, $pf$ denotes the fibre's packing fraction. It is self-evident that ${v}_{RUC}^{NCF}+{v}_{RUC}^{NC}=1,$ and it is also evident that hexagonal packing is used to calculate packing fraction. ${v}_{RUC}^{NCF}$ from Eq. (62) and iso-field condition in conduction with rules of mixture the compliance, ${{S}{\prime}}_{w}$ can be evaluated considering the axis of transverse isotropy along the axis of the wrap yarn. After appropriate transformation and orientation averaging can be applied on ${{S}{\prime}}_{w}$ to get the averaged compliance ${S}_{\overline{w} }$ as follows.

63 $${S}_{\overline{w} }=\frac{1}{\beta }\underset{0}{\overset{{\beta }_{cw}}{\int }}{T}_{r2}^{t}{{S}{\prime}}_{w}{T}_{r2}^{t}d\beta$$

The transformation matrices, ${T}_{r2}$ has been considered to transform ${{S}{\prime}}_{w}$ about the axis ‘2’. In the Eq. (63), the individual terms of transformed compliance ${S}_{\overline{w} }$ are as follows:

64 $$\begin{gathered} S_{{\bar{w}}} \left( {1,1} \right) = \frac{{\left\{ {A_{{\bar{w}}} S^{\prime } _{{11}} + B_{{\bar{w}}} S^{\prime } _{{33}} + C_{{\bar{w}}} \left( {S^{\prime } _{{13}} + S^{\prime } _{{31}} + S^{\prime } _{{55}} } \right)} \right\}}}{{\beta _{{cw}} }};S_{{\bar{w}}} \left( {1,2} \right) = \frac{{\left( {D_{{\bar{w}}} S^{\prime } _{{12}} + E_{{\bar{w}}} S^{\prime } _{{32}} } \right)}}{{\beta _{{cw}} }}; \hfill \\ S_{{\bar{w}}} \left( {1,3} \right) = \frac{{\left\{ {A_{{\bar{w}}} S^{\prime } _{{13}} + B_{{\bar{w}}} S^{\prime } _{{31}} + C_{{\bar{w}}} \left( {S^{\prime } _{{11}} + S^{\prime } _{{33}} - S^{\prime } _{{55}} } \right)} \right\}}}{{\beta _{{cw}} }};S_{{\bar{w}}} \left( {2,3} \right) = \frac{{\left( {E_{{\bar{w}}} S^{\prime } _{{21}} + D_{{\bar{w}}} S^{\prime } _{{23}} } \right)}}{{\beta _{{cw}} }}; \hfill \\ S_{{\bar{w}}} \left( {1,5} \right) = \frac{{\left\{ {F_{{\bar{w}}} \left( {S^{\prime } _{{13}} - S^{\prime } _{{11}} } \right) + G_{{\bar{w}}} \left( {S^{\prime } _{{31}} - S^{\prime } _{{33}} } \right) + H_{{\bar{w}}} S^{\prime } _{{55}} } \right\}}}{{\beta _{{cw}} }};S_{{\bar{w}}} \left( {2,5} \right) = \frac{{2I_{{\bar{w}}} \left( {S^{\prime } _{{21}} - S^{\prime } _{{23}} } \right)}}{{\beta _{{cw}} }}; \hfill \\ S_{{\bar{w}}} \left( {2,2} \right) = S\prime _{{22}} ;S_{{\bar{w}}} \left( {3,3} \right) = \frac{{\left\{ {B_{{\bar{w}}} S^{\prime } _{{11}} + A_{{\bar{w}}} S^{\prime } _{{33}} + C_{{\bar{w}}} \left( {S^{\prime } _{{13}} + S^{\prime } _{{31}} + S^{\prime } _{{55}} } \right)} \right\}}}{{\beta _{{cw}} }}; \hfill \\ S_{{\bar{w}}} \left( {3,5} \right) = \frac{{\left\{ {F_{{\bar{w}}} \left( {S^{\prime } _{{33}} - S^{\prime } _{{31}} } \right) + G_{{\bar{w}}} \left( {S^{\prime } _{{11}} - S^{\prime } _{{13}} } \right) + H_{{\bar{w}}} S\prime _{{55}} } \right\}}}{{\beta _{{cw}} }}; \hfill \\ S_{{\bar{w}}} \left( {4,4} \right) = \frac{{\left( {D_{{\bar{w}}} S^{\prime } _{{44}} + E_{{\bar{w}}} S^{\prime } _{{66}} } \right)}}{{\beta _{{cw}} }};S_{{\bar{w}}} \left( {4,6} \right) = \frac{{I_{{\bar{w}}} \left( {S^{\prime } _{{66}} - S^{\prime } _{{44}} } \right)}}{{\beta _{{cw}} }}; \hfill \\ S_{{\bar{w}}} \left( {5,5} \right) = \frac{{\left\{ {J_{{\bar{w}}} \left( {S^{\prime } _{{11}} - S^{\prime } _{{13}} - S^{\prime } _{{31}} + S^{\prime } _{{33}} } \right) + K_{{\bar{w}}} S^{\prime } _{{55}} } \right\}}}{{\beta _{{cw}} }}; \hfill \\ S_{{\bar{w}}} \left( {6,6} \right) = S^{\prime } _{{\bar{w}}} \left( {4,4} \right) \hfill \\ \end{gathered}$$

Appendix D contains a list of the associated terms in Eq. (64). Individual terms of ${S}_{\overline{f} }$ are also computed in the same way by transforming and averaging ${{S}{\prime}}_{f}$ about axis ‘1’. The wrap yarn's effective elastic compliance matrix ${S}_{W}$ may be determined by

65 $${S}_{W}={\mu }_{cw}{S}_{\overline{w} }+\left(1-{\mu }_{cw}\right){{S}{\prime}}_{\overline{w} }$$

Volume averaging may be used to evaluate the RUC's effective in-plane stiffness (${C}_{RUC}^{I}$) and out of plane compliance (${S}_{RUC}^{O}$).

66 $$\begin{array}{c}{S}_{RUC}^{O}={S}_{W}\left(\frac{{V}_{yw}}{{V}_{yf}+{V}_{yw}}\right)+{S}_{F}\left(\frac{{V}_{yf}}{{V}_{yf}+{V}_{yw}}\right)\\ {C}_{RUC}^{I}={C}_{W}\left(\frac{{V}_{yw}}{{V}_{yf}+{V}_{yw}}\right)+{C}_{F}\left(\frac{{V}_{yf}}{{V}_{yf}+{V}_{yw}}\right)\end{array}$$

where ${C}_{k}$ is the inverse of ${S}_{k}$ by taking $k=W\text{\hspace{0.17em}}\text{and\hspace{0.17em}}F$. Finally, the effective elastic properties from Eq. (66) are obtained as follows.

67 $$\begin{gathered} E_{1} = \frac{1}{{S_{{RUC}}^{I} \left( {1,1} \right)}};\quad E_{2} = \frac{1}{{S_{{RUC}}^{I} \left( {2,2} \right)}};\quad E_{3} = \frac{1}{{S_{{RUC}}^{O} \left( {3,3} \right)}}; \hfill \\ G_{{12}} = \frac{1}{{S_{{RUC}}^{I} \left( {6,6} \right)}};\quad G_{{23}} = \frac{2}{{\left\{ {S_{{RUC}}^{O} \left( {4,4} \right) + S_{{RUC}}^{I} \left( {4,4} \right)} \right\}}};\quad G_{{31}} = \frac{2}{{\left\{ {S_{{RUC}}^{O} \left( {5,5} \right) + S_{{RUC}}^{I} \left( {5,5} \right)} \right\}}} \hfill \\ \nu _{{12}} = \frac{{ - S_{{RUC}}^{O} \left( {1,2} \right)}}{{S_{{RUC}}^{I} \left( {1,1} \right)}};\quad \nu _{{23}} = \frac{{ - S_{{RUC}}^{O} \left( {3,2} \right)}}{{S_{{RUC}}^{I} \left( {3,3} \right)}}; \hfill \\ \end{gathered}$$

## Results and discussions

A MATLAB programme has been developed for material modelling based on mathematical formulas discussed in the previous sections. Based on the formulations, the composite material system, as represented in Fig. 1, has three basic sub-material systems such as 1. *NC* phase (modified matrix) formed by adding *CNT* and polymer, 2. Yarn formed by *NC* phase and fibre, and finally, 3. Combination of undulated yarn and polymer phase (*RUC*). The proposed micromechanical framework needs validation at the sub-component level; hence, three validation stages were adopted, where the first two validation stages were conducted for the *NC* phase with and without agglomeration. The second phase of validation was carried out for yarns and *RUC.* After the validation, the novel micromechanics was utilised for further studies.

### Material property validation

**Fig. 7:**
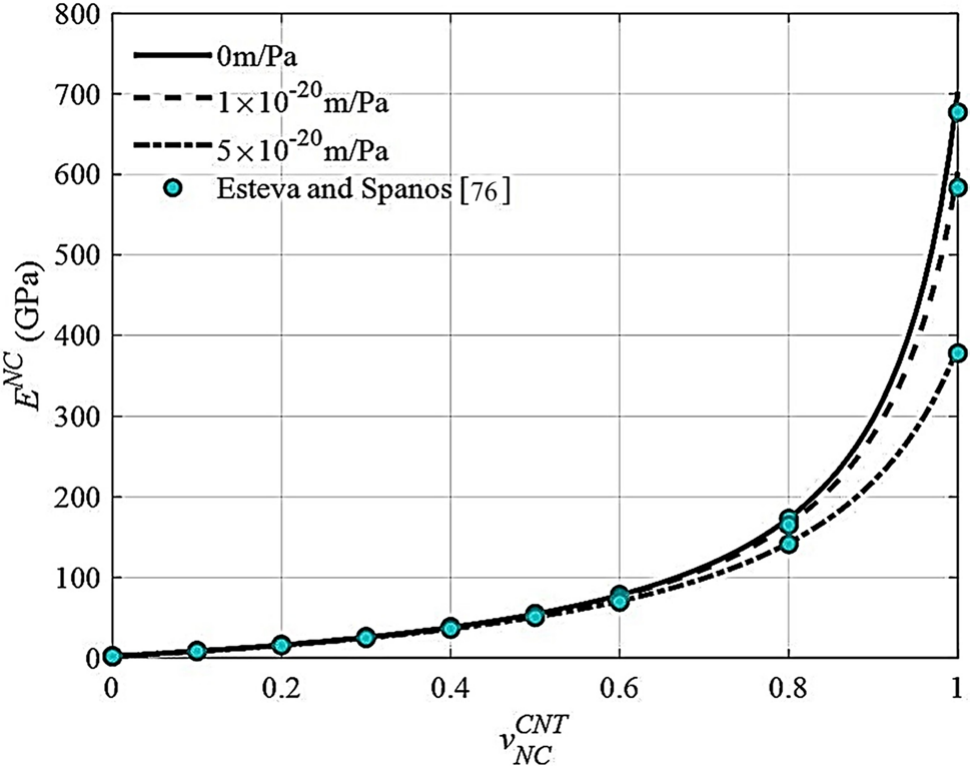
Young’s modulus variation with carbon nanotubes for different interfacial compliance

The elastic properties of nanocomposite for various interfacial compliance values were investigated using Eq. (43), as illustrated in Fig. 7. The material parameters of the nanocomposite constituents used in this validation stage were obtained from Ref. [76, 79]. The Young's modulus of nanocomposites found is quite similar to Esteva and Spanos’ findings [76], as shown in Fig. 7 where ${l}_{cnt}/{d}_{cnt}$ is equal to 50 and ${d}_{cnt}$ is considered as 1.7 nm.

**Fig. 8:**
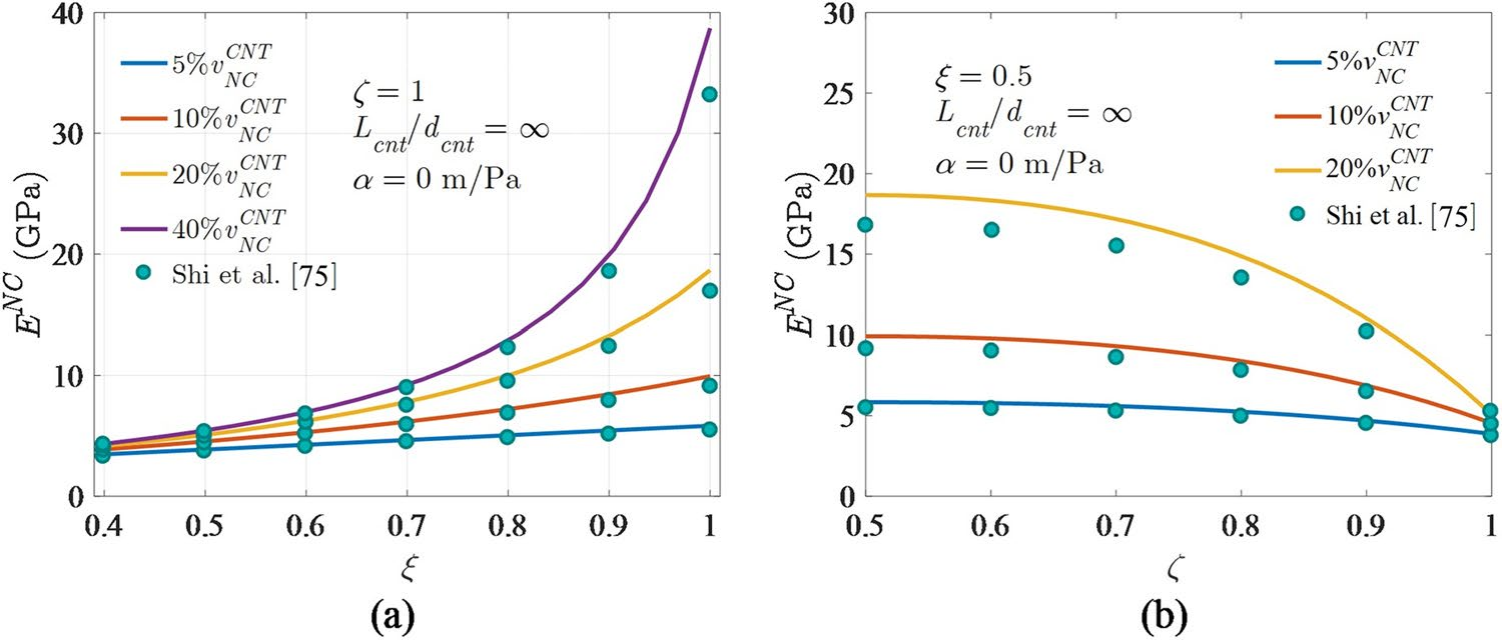
Young’s modulus variation with agglomeration parameters **a** $\zeta$ and **b** $\xi$ for different CNT content

Another validation is carried out to assess the correctness of the suggested formulation based on published nanocomposite [75] results when agglomeration is considered. The CNTs are assumed to be entirely bound to the matrix in this case (i.e.. $\alpha =0$)*.* This validation's attributes are also obtained from Ref. [75], with the ${l}_{cnt}/{d}_{cnt}$ value being ∞. Figure 8a, b demonstrate that the proposed formulation can produce outstanding results similar to those reported by Shi et al. [75].The proposed formulation, as shown in Fig. 8a, b, can produce outstanding results that are similar to those achieved by Shi et al. [75]. The negligible variance may be due to matrix manipulation rather than the explicit expression offered by Shi et al. [75].

### Validation of elastic characteristics

Plane woven composite material, which consists of carbon fibre as reinforcement and aluminium metal as the matrix, was used to validate the formulations, and the results are shown in Table 1. Table 1 illustrates that the findings are in accordance with prior study published in Ref. [86].

**Table 1:** Comparison of Geometric parameters and mechanical properties

Work →	Lee et al. [86]		Present
Parameter ↓	Theoretical	Experimental	
${E}_{1}$ (GPa)	98.9	95.0	94.3042
${E}_{2}$ (GPa)	92.8	90.7	88.0000
${E}_{3}$ (GPa)	62.1	58.0	56.1766
${G}_{12}$ (GPa)	25.2	22.6	25.2349
${G}_{23}$ (GPa)	23.0	20.7	22.3611
${G}_{31}$ (GPa)	24.4	19.5	22.8255
${\nu }_{12}$	0.24	0.21	0.2307
${\nu }_{23}$	0.43	0.40	0.4054
${\nu }_{31}$	0.20	0.20	0.2148
${\beta }_{cw}$ (Degree)	7.00	7.54	7.0037
${\beta }_{cf}$ (Degree)	7.46	7.78	7.4650
${L}_{w}$ (mm)	2.35	2.73	2.3407
${L}_{f}$ (mm)	2.63	2.72	2.6213
${v}_{RUC}$ (% age)	35.1	37.0	35.16

### Viscoelastic properties of individual constituents of material system

**Fig. 9:**
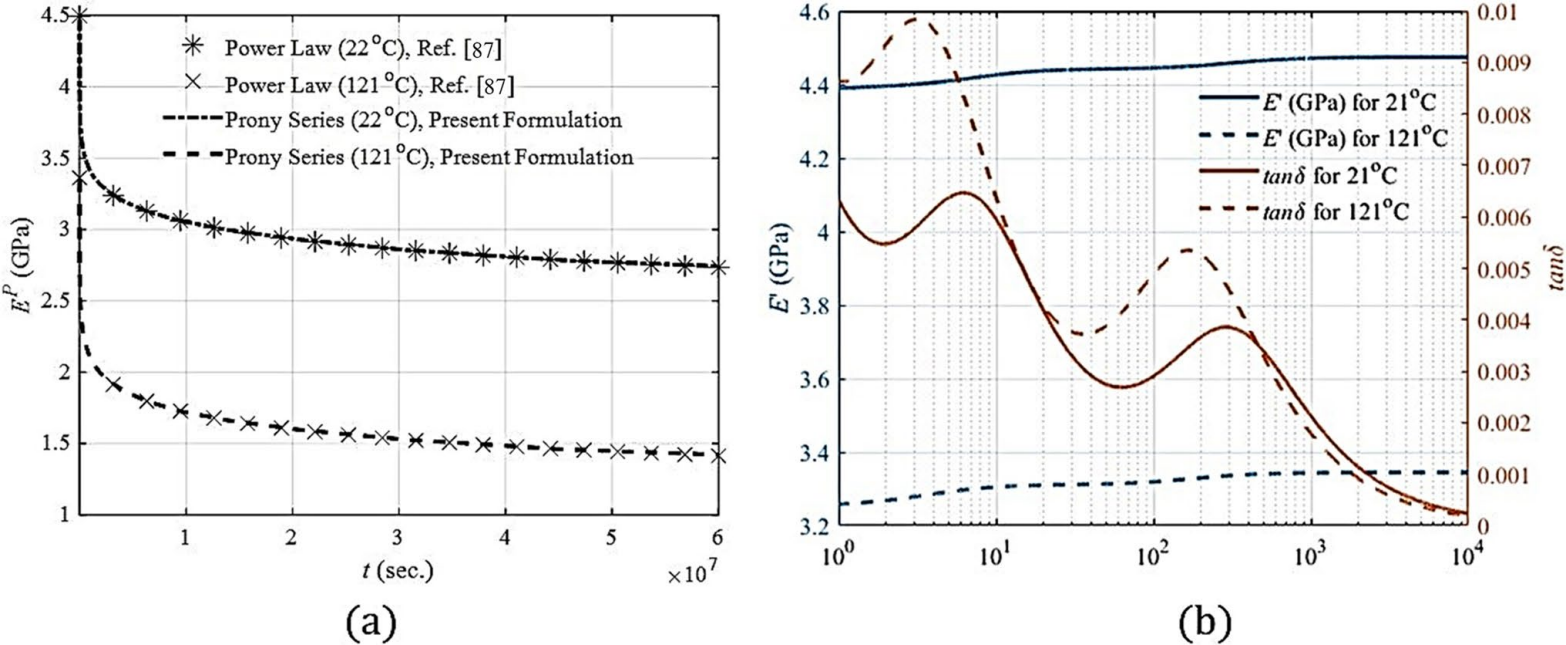
**a** Power Law and the Prony series fit of the relaxation modulus **b** Storage modulus and loss factor for 934 Epoxy

934 Epoxy has been chosen as the matrix material for which the creep compliance was modelled with a power-law model as ${M}^{P}(t)={D}_{0}+{D}_{1}{t}^{n}$ and individual parameters of the model for different temperatures have been taken from Ref. [87]. The stress relaxation modulus (${E}^{P}(t)$) has been numerically estimated with sufficient accuracy using eleven Prony series parameters, as illustrated in Fig. 9a. The parameters for the Prony series are also listed in Table 2. By utilising these parameters of the Prony series, storage modulus and loss factor for 934 Epoxy were evaluated, which are shown in Fig. 9b. Figure 9a demonstrates a decline in the relaxation modulus of the polymer as time and temperature rise, whereas Fig. 9b indicates an increase in the storage modulus with higher frequencies. Conversely, when the temperature rises, there is a corresponding drop. Consequently, the polymer exhibits a glassy behavior at higher frequencies and lower temperatures, whereas it displays a lathery behavior at lower frequencies and higher temperatures. The influence of time and frequency on the viscoelastic characteristics of the polymer is evident in Fig. 9a, b, whereas the thermoelastic properties of the polymer exhibit a gradual and more mild effect. Other constituent material properties (such as SWCNTs (10,10) and carbon fibre) are used from the available literature [86, 88–92] and treated as perfectly elastic in the current studies.

**Table 2:** Coefficients of Prony series for 934 Epoxy [87]

Temperature	22 °C		121 °C	
$i$	${E}_{i}^{P}$	${\tau }_{i}^{P}$	${E}_{i}^{P}$	${\tau }_{i}^{P}$
∞	2,662,987,567.34533		1,349,975,132.53024	
1	31,946,874.76110	0.00318	33,099,418.36941	0.00555
2	49,018,280.61009	0.14131	54,921,781.93832	0.27136
3	71,104,573.33824	3.45918	84,128,339.35263	6.45715
4	98,525,506.12263	57.28423	120,697,032.39106	99.30141
5	131,244,467.15976	718.90630	163,298,090.01449	1135.21594
6	168,295,436.39346	7329.32500	208,326,889.33604	10,457.53608
7	207,787,173.05179	63,467.54972	250,443,122.02547	81,746.19867
8	247,382,936.60221	482,907.43713	283,717,854.54735	563,853.53571
9	294,344,495.42045	3,385,519.05366	314,527,790.33682	3,616,027.08768
10	511,764,382.06722	31,518,411.26350	481,410,103.45771	30,734,238.59700

### Problem specifications

The effects of several parameters on viscoelastic storage moduli and loss factors have been explored by varying CNTs volume fractions (${v}_{NC}^{CNT}$), non-dimensional CNTs-polymer interphase compliance (${\alpha }_{0}$), CNTs- polymer interphase loss factor (${\alpha }^{^{\prime\prime} }/{\alpha }{\prime}$), agglomeration parameters ($\zeta$ and $\xi$), the aspect ratio of CNTs (${l}_{cnt}/{d}_{cnt}$), and temperature ($T$ in ^°^C) and excitation frequency ($\omega$), the thickness of yarn (${t}_{f}$), the width of yarn (${S}_{Ff}{t}_{f}$), the ratio of gap length to width of yarn (${L}_{gw}/{S}_{Ff}{t}_{f}$) and the fibre packing factor ($pf$). The range of the variation of parameters is listed in Table 3.ffects of carbon nanotube content (${{\varvec{v}}}_{{\varvec{N}}{\varvec{C}}}^{{\varvec{C}}{\varvec{N}}{\varvec{T}}}$) and fibre content (${\varvec{p}}{\varvec{f}}$) on the viscoelastic properties.

**Table 3:** Variation of parameters

Subsections	${v}_{NC}^{CNT}$ (%)	$pf$ (%)	$\frac{{l}_{cnt}}{{d}_{cnt}}$	${\alpha }_{0}$	$\frac{\alpha {\prime}{\prime}}{\alpha {\prime}}$	$\zeta$	$\xi$	$T$ ( ^o^C)	$\omega$ (rad/sec)	${S}_{Ff}{t}_{f}={S}_{Fw}{t}_{w}$ (mm)	${t}_{f}={t}_{w}$ (mm)	$\frac{{L}_{gw}}{{S}_{Ff}{t}_{f}}=\frac{{L}_{gf}}{{S}_{Fw}{t}_{w}}$
3.4.1	V	V	50	1.2	0.2	0.7	0.5	22	10	6	0.6	0.4
3.4.2	V	60	V	1.2	0.2	0.7	0.5	22	10	6	0.6	0.4
3.4.3	V	60	50	V	0.2	0.7	0.5	22	10	6	0.6	0.4
3.4.4	V	60	50	1.2	V	0.7	0.5	22	10	6	0.6	0.4
3.4.5	V	60	50	1.2	0.2	V	0.5	22	10	6	0.6	0.4
3.4.6	V	60	50	1.2	0.2	0.7	V	22	10	6	0.6	0.4
3.4.7	20	60	50	1.2	0.2	0.7	0.5	V	V	6	0.6	0.4
3.4.8	20	60	50	1.2	0.2	0.7	0.5	22	10	V	V	0.4

V suggest variation in the parameter

#### Effects of CNT conten (${{\varvec{v}}}_{{\varvec{N}}{\varvec{C}}}^{{\varvec{C}}{\varvec{N}}{\varvec{T}}}$) and packing factor (***pf***) on the viscoelastic properties

**Fig. 10:**
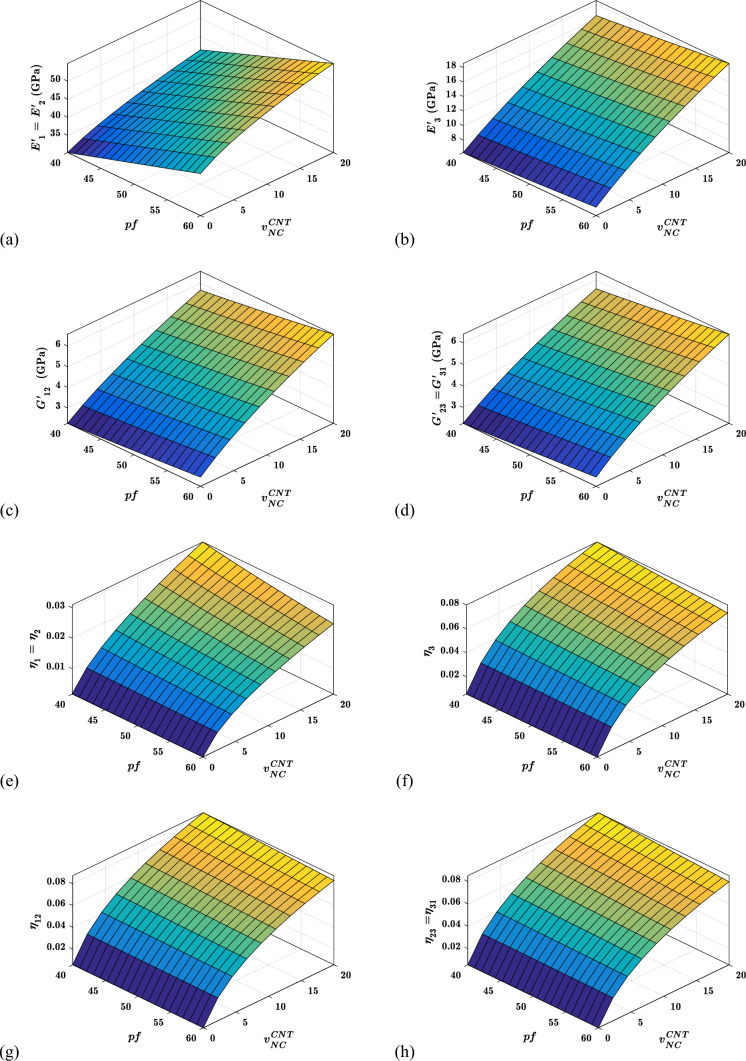
Variation of the viscoelastic properties with ${v}_{NC}^{CNT}$ and $pf$

The viscoelastic property of suggested hybrid nanocomposite materials for different fibre packing factors and CNTs concentrations are shown in Fig. 10. The in-plane and out-of-plane storage moduli improve with the fibre packing factor and CNTs content, as shown in Fig. 10a–d. The increase in the content of CNTs and fibres in the RUC is responsible for improving storage moduli. The in-plane storage moduli ${E}_{1}{\prime}$ and ${E}_{2}{\prime}$ are significantly more impacted by the CNTs content ${v}_{NC}^{CNT}$ and packing factor $pf$, than other storage moduli, as shown in Fig. 10a–d. In contrast, the in-plane and out-of-plane loss factors improve with a rise in CNTs content ${v}_{NC}^{CNT}$. But worsen with an increase in fibre packing factor $pf$, as shown in Fig. 10e–h. This is because of an increase in the $pf$ causes the $NC$ phase to be squeezed out of the warp and fill yarn. The $NC$ phase improves the RUC's damping performance. As the volume of the $NC$ phase in both yarns decreases, the loss factors have deteriorated with the increase in $pf$.

#### Effects of aspect ratio (${{\varvec{l}}}_{{\varvec{c}}{\varvec{n}}{\varvec{t}}}/{{\varvec{d}}}_{{\varvec{c}}{\varvec{n}}{\varvec{t}}}$) of CNTs on the viscoelastic properties

**Fig. 11:**
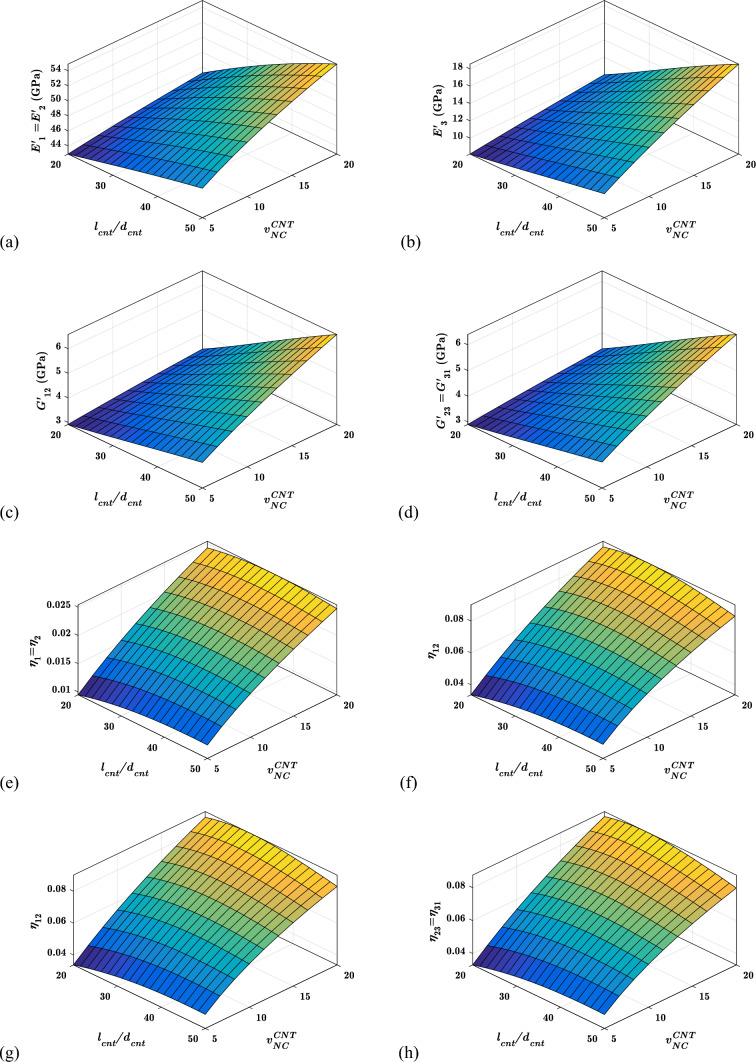
Variation of the viscoelastic properties with ${v}_{NC}^{CNT}$ and ${l}_{cnt}/{d}_{cnt}$

For various aspect ratios of CNTs (${l}_{cnt}/{d}_{cnt}$) and CNTs concentrations, Fig. 11 shows the viscoelastic properties of the proposed hybrid nanocomposite material system. The addition of high aspect ratio CNTs improves all in-plane and out-of-plane storage moduli, as shown in Fig. 11a–d.

The rationale for this phenomenon is that longer CNTs can have stronger CNTs-polymer contact, resulting in higher stress transfer between the two separate phases due to their high aspect ratio. However, as shown in Fig. 11e–h, the in-plane and out-of-plane loss factors do not necessarily rise as the aspect ratio increases. For lower-value CNTs contents, the loss factor increases with increasing aspect ratio and remains constant. When the CNTs content is raised, the suggested material system becomes highly stiff and glassy, lowering the loss factors after a specific aspect ratio due to the mentioned phenomenon. However, regardless of the aspect ratio of the CNTs, it is easy to see that as the CNTs content increases, all loss factors improve. Experiments by de Borbón et al. [93] and micromechanical analysis by Finegan et al. [94] came to similar conclusions about the impacts of aspect ratio.

#### Effects of interfacial compliance (${\boldsymbol{\alpha }}_{0}$) on the viscoelastic properties

**Fig. 12:**
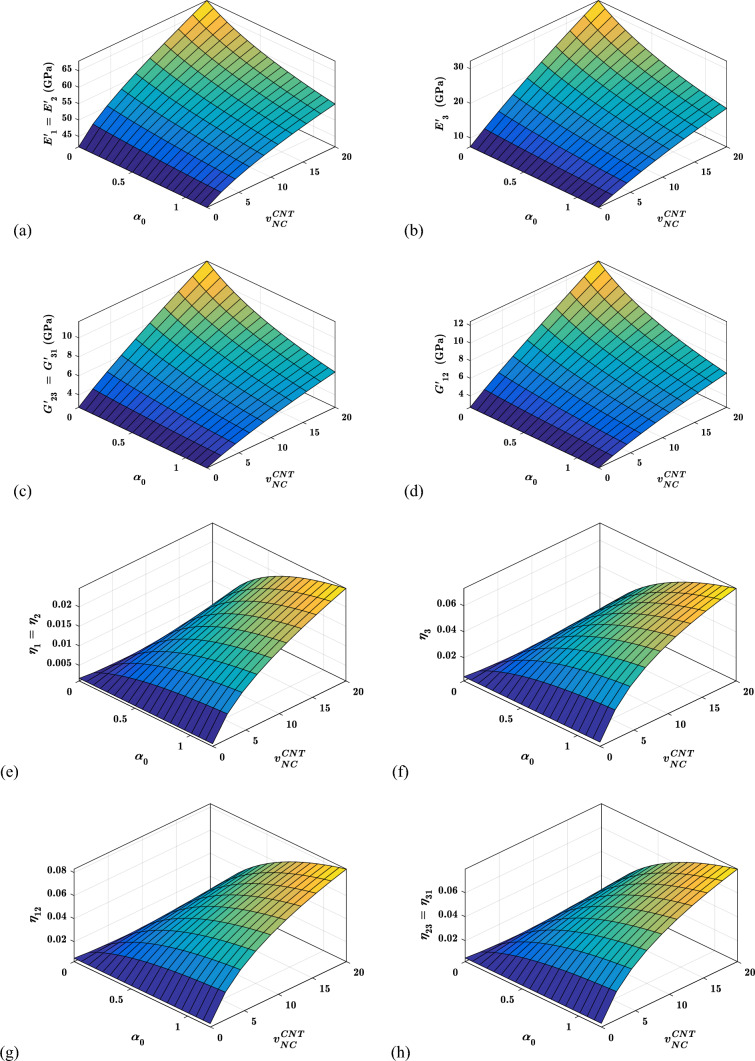
Variation of the viscoelastic properties with ${\alpha }_{0}$ and ${v}_{NC}^{CNT}$

Figure 12 shows the influence of non-dimensional interfacial compliance ($${\alpha }_{0}$$) and carbon nanotube content ($${v}_{NC}^{CNT}$$) on the viscoelastic characteristics of hybrid nanocomposites. Figure 12a–d show that storage moduli fall as interfacial compliance ($${\alpha }_{0}$$) increases, which is understandable given the fact that when CNTs-polymer interfacial compliance increases in the $$NC$$ phase, the material system becomes more flexible regardless of CNTs content ($${v}_{NC}^{CNT})$$. Figure 12e–h show that as interfacial compliance ($${\alpha }_{0}$$) increases, all loss factors increase, regardless of CNTs content ($${v}_{NC}^{CNT})$$. This is because increasing, $${\alpha }_{0}$$ promotes CNTs movement inside the polymer phase, which aids energy dissipation. At lower $${v}_{NC}^{CNT}$$, the effect of interfacial compliance ($${\alpha }_{0}$$) on the viscoelastic property is more pronounced.

#### Effects of interfacial loss factor (${\boldsymbol{\alpha }}^{^{\prime\prime} }/{\boldsymbol{\alpha }}^{\boldsymbol{^{\prime}}}$) on the viscoelastic properties

**Fig. 13:**
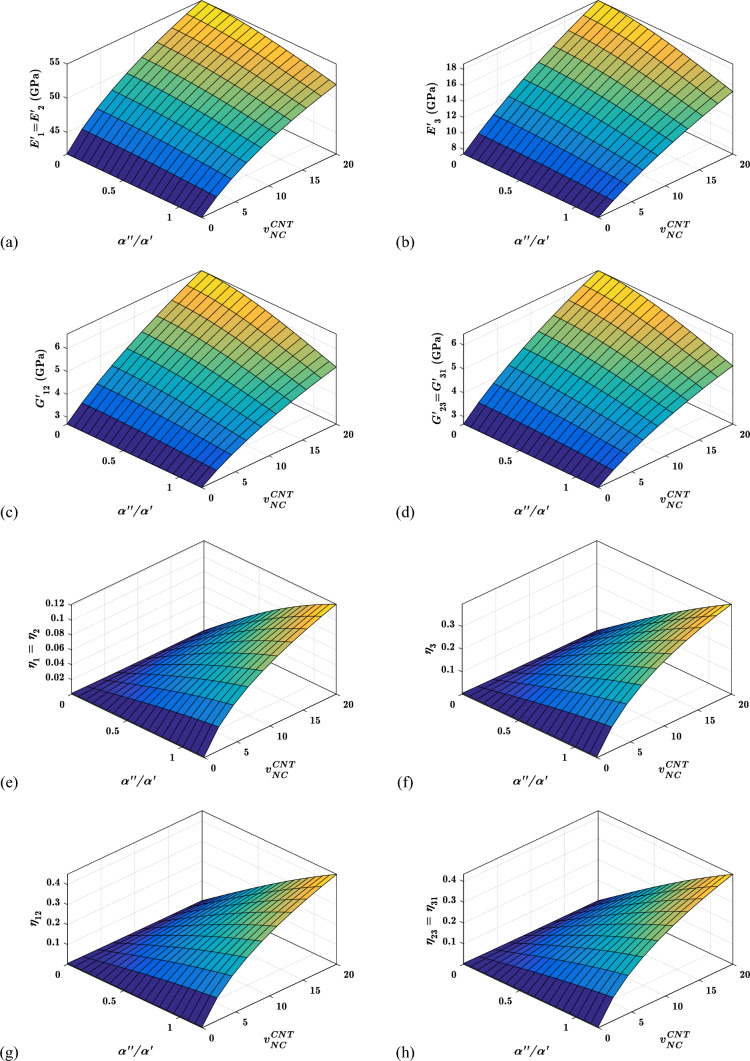
Variation of the viscoelastic properties with ${\alpha }^{^{\prime\prime} }/{\alpha }^{\prime}$ and ${v}_{NC}^{CNT}$

Figure 13 illustrates the relationship between the interfacial loss factor (${\alpha }^{^{\prime\prime} }/{\alpha }{\prime}$) and the carbon nanotube content (${v}_{NC}^{CNT}$) on the viscoelastic properties of multi-scale hybrid nanocomposites. The storage moduli depicted in Fig. 13a–d exhibit a notable decrease as ${\alpha }^{^{\prime\prime} }/{\alpha }{\prime}$ increases. This decline suggests that the interfacial storage modulus plays a crucial role in rendering the multiscale composite flexible and leathery (or rubbery), irrespective of ${v}_{NC}^{CNT}$. Moreover, as the ${v}_{NC}^{CNT}$ increases, the influence of ${\alpha }^{^{\prime\prime} }/{\alpha }{\prime}$ on the storage moduli becomes more pronounced.

In Fig. 13e–h, it is evident that all loss factors exhibit an ascendant trend with an increase in interfacial loss factor (${\alpha }^{^{\prime\prime} }/{\alpha }{\prime}$), regardless of nanotube content (${v}_{NC}^{CNT}$). This trend is attributed to the simultaneous rise in both interfacial and overall damping properties of the proposed multiscale hybrid composite material system. Essentially, as the interfacial damping property increases, it contributes to an overall increase in the damping capability of the hybrid composite material. This observation emphasizes the significant role of interfacial interactions between polymer and carbon nanotube in dictating the viscoelastic behaviour of nanocomposite materials.

#### Effects of agglomeration parameter (${\varvec{\zeta}}$)on the viscoelastic properties

**Fig. 14:**
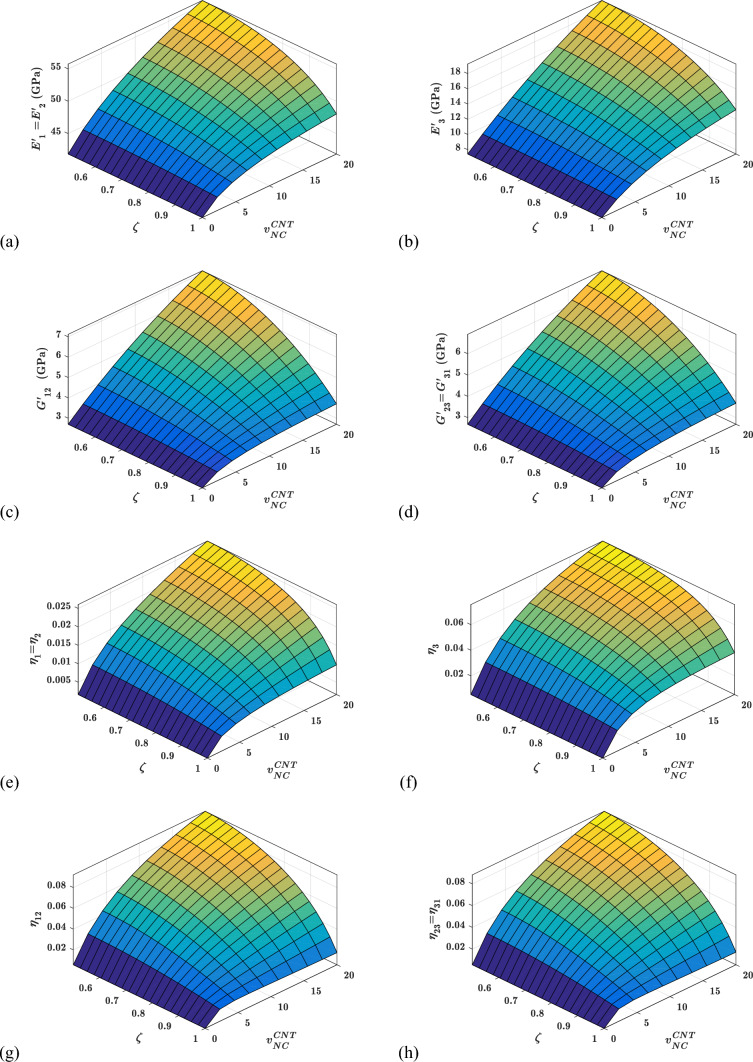
Variation of the viscoelastic properties with $\zeta$ and ${v}_{NC}^{CNT}$

The viscoelastic characteristics of the proposed woven fabric hybrid nanocomposite for various agglomeration parameters ($\zeta$) and CNTs content (${v}_{NC}^{CNT}$) are shown in Fig. 14. Figure 14 shows that as the agglomeration parameter ($\zeta$) is increased, all of the storage moduli and loss factors degrade. Figure 14 further shows that as ${v}_{NC}^{CNT}$ increases, the influence of $\zeta$ on storage moduli and loss factors increases. After approximately 5%, ${v}_{NC}^{CNT}$ becomes ineffective in terms of improving all loss moduli at a higher value of $\zeta$. As $\zeta ={V}_{cluster}^{CNT}/{V}^{CNT}$, a rise in $\zeta$ will ensure an increase in the volume of CNTs in the cluster. CNTs outside the cluster diminish as the number of CNTs or volume of CNTs in the cluster grows. The volume outside the cluster is substantial, and the population of CNTs is significantly lower than the population of CNTs within the cluster. Because the volume of inclusions is so relatively small in relation to the volume outside of the inclusion in an RVE, the material property outside the cluster always dominates the viscoelastic characteristics of the proposed hybrid composite. As a result, increasing $\zeta$ deteriorates both elastic and damping properties.

#### Effects of agglomeration parameter (${\varvec{\xi}}$) on the viscoelastic properties

**Fig. 15:**
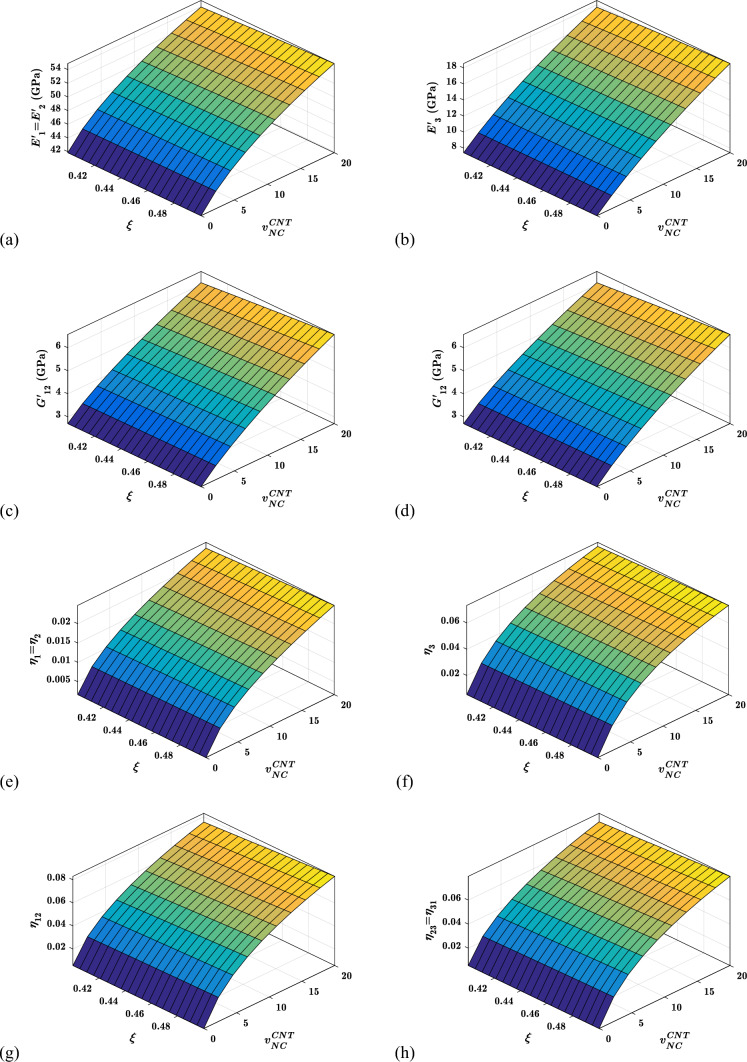
Variation of the viscoelastic properties with $\xi$ and ${v}_{NC}^{CNT}$

The viscoelastic properties of the proposed hybrid woven-fabric nanocomposite material for various agglomeration parameters ($\xi$) and CNTs contents (${v}_{NC}^{CNT}$) are shown in Fig. 15. Figure 15 shows that increasing $\xi$ improves all storage moduli and loss factors of the proposed material system. By definition, $\xi ={V}^{cluster}/{V}^{NC}$ (i.e. the volume fraction of the cluster with respect to the nanocomposite) so the volume of clusters inside the nanocomposite grows as $\xi$ increases. Because this cluster may be thought of as a reinforcing phase in the material system, increasing $\xi$ will increase the volume of clusters with respect to the nanocomposite. So increase in $\xi$ enhances the storage and loss factor of the inclusion. As a result, the NC's overall attributes have improved.

#### Effects of temperature (${\varvec{T}}$) and excitation frequency (${\varvec{\omega}}$) on the viscoelastic properties

**Fig. 16:**
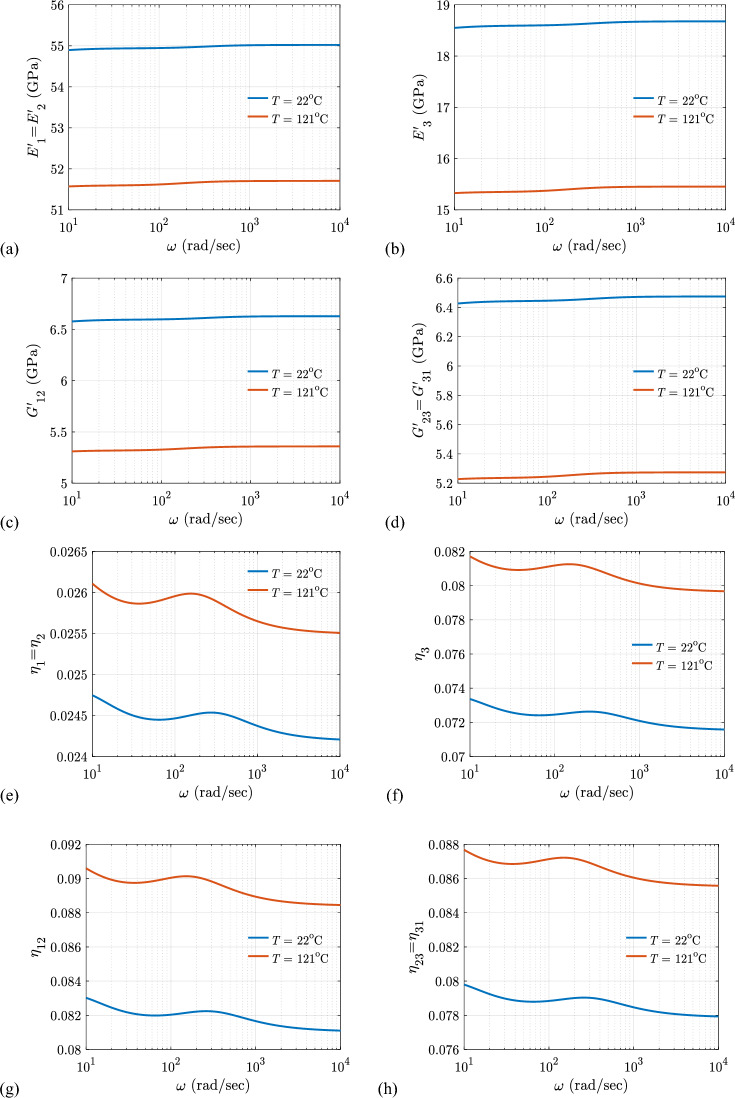
Variation of the viscoelastic properties with $T$ and $\omega$

Figure 16 shows the changes in viscoelastic properties of proposed material systems as a function of temperature ($T$) and excitation frequency ($\omega$). Figure 16 illustrates that temperature and excitation frequency have a considerable impact on the viscoelastic properties of the said material system. All the storage moduli increases as the excitation frequency ($\omega$) increase, as seen in Fig. 16a–d. According to the matrix property, the loss factors vary with excitation frequency, as shown in Fig. 16e–h. This means that the hybrid composite can act glassy and rubbery at a given frequency regardless of temperature. The proposed composite material systems have lower storage moduli and larger loss moduli at higher temperatures, as shown in Fig. 16, as at lower temperatures, the polymer is more glassy.

#### Effects of the thickness of the yarn (${{\varvec{t}}}_{{\varvec{f}}}$) and width of yarn (${{\varvec{S}}}_{{\varvec{F}}{\varvec{f}}}{{\varvec{t}}}_{{\varvec{f}}}$) on the viscoelastic properties

**Fig. 17:**
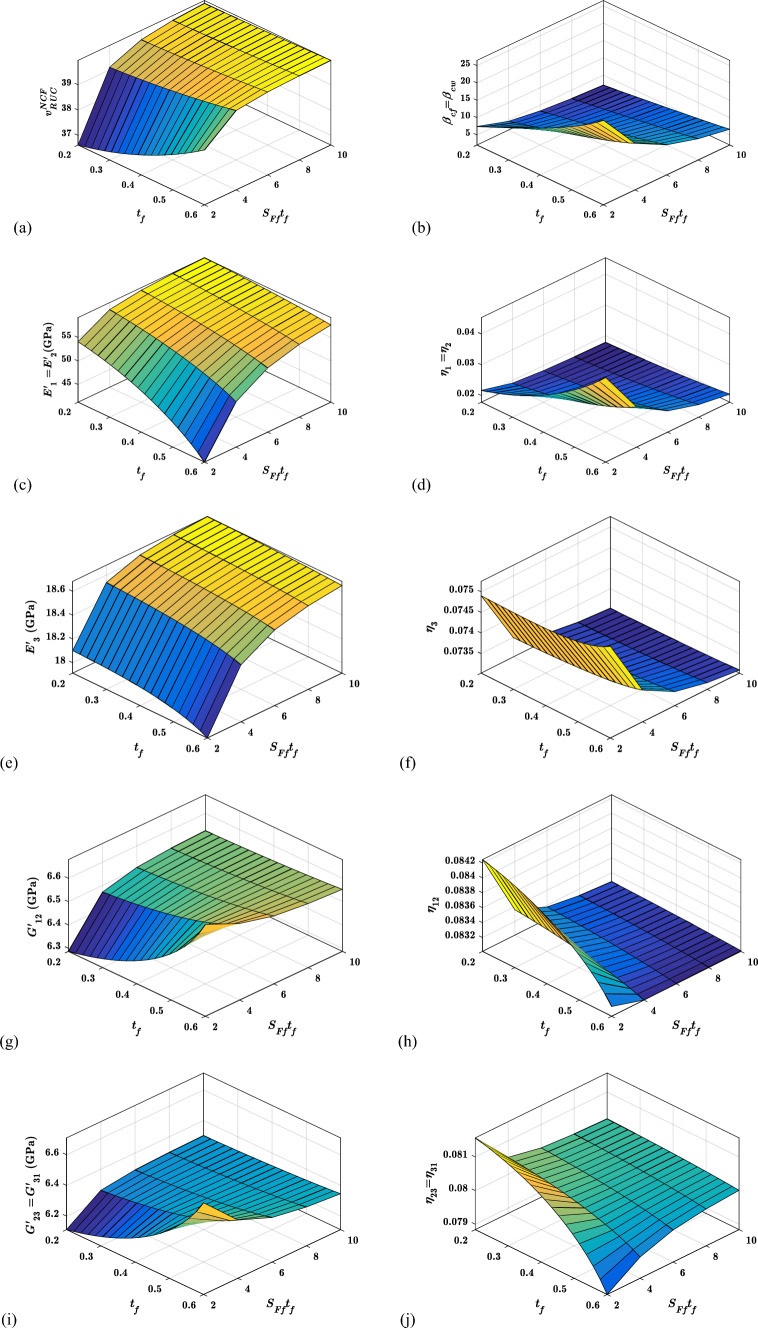
Variation of the viscoelastic properties with ${S}_{Ff}{t}_{f}$ and ${t}_{f}$

Figure 17 represents the viscoelastic properties of the proposed woven fabric hybrid nanocomposite for the various width of yarn ($${S}_{Ff}{t}_{f}$$) and thickness of the yarn $$({t}_{f})$$. Figure 17a, b demonstrate the variations of the volume fraction of fibre in $$RUC$$ ($${v}_{RUC}^{NCF}$$) and crimp angle ($${\beta }_{cf}$$) with respect to fill-yarn thickness ($${t}_{f})$$ and width of fill yarn ($${S}_{Ff}{t}_{f}$$). Figure 17a shows that when the yarn thickness ($${t}_{f}$$) increase, so does the value of $${v}_{RUC}^{NCF}$$. for the lower value of yarn width ($${S}_{Ff}{t}_{f}$$) how ever at higher levels of yarn width ($${S}_{Ff}{t}_{f}$$) there is no substantial fluctuation in $${v}_{RUC}^{NCF}$$ with regard to yarn thickness ($${t}_{f}$$). These effets are seen as yarn thickness ($${t}_{f}$$) increases the volume fraction of $$NCF$$ phase in the $$RUC$$ for a certain breadth of yarn ($${S}_{Ff}{t}_{f}$$). In the instance of crimp angle ($${\beta }_{cf}$$) variations as illustrated in Fig. 17b, it can be seen that the crimp angle ($${\beta }_{cf}$$) increase as yarn thickness ($${t}_{f}$$) increases; on the other hand, it reduces with the increase in yarn width ($${S}_{Ff}{t}_{f}$$). When the section e-f-g-h in Fig. 6 is taken into consideration, the crimp angle ($${\beta }_{cf}$$) increases as the yarn thickness ($${t}_{f}$$) increases. However, when sections a-b-c-d and e-f-g-h, as illustrated in Fig. 6, are considered, it can be ascertained that for a fixed thickness of yarn ($${t}_{f}$$), increasing the width of the yarn ($${S}_{Ff}{t}_{f}$$) will result in a decrease in crimp angle.

Figure 17c shows the variation of in-pane storage modulus (${E}_{1}{\prime}$) with respect to yarn thickness (${t}_{f}$) and yarn width (${S}_{Ff}{t}_{f}$). It is clear that the in-pane storage modulus (${E}_{1}{\prime}$) improves as the yarn width (${S}_{Ff}{t}_{f}$) increases. However, irrespective of the value of yarn width (${S}_{Ff}{t}_{f}$), the in-pane storage modulus (${E}_{1}{\prime}$) deteriorates with yarn thickness (${t}_{f}$). As a result, it can be stated that increasing yarn thickness (${t}_{f}$) has almost no effect on the in-pane storage modulus (${E}_{1}{\prime}$) however, it can be stated that the width of the yarn (${S}_{Ff}{t}_{f}$) significantly improves the in-pane storage modulus (${E}_{1}{\prime}$). The fluctuation of the in-plane loss factor (${\eta }_{1}$) in Fig. 17d demonstrates that it follows the trend of crimp angle (${\beta }_{cf}$). It means with the increase in crimp angle (${\beta }_{cf}$) the proposed hybrid nanocomposite material system behaves as more rubbery or leathery when it is stretched (or compressed) periodically in direction 1 (or 2)

The variation of out–of–plane storage modulus ($${E}_{3}{\prime}$$) with yarn thickness ($${t}_{f})$$ and yarn width ($${S}_{Ff}{t}_{f}$$) is shown in Fig. 17e. It is evident from Fig. 17c, e that the out–of–plane storage modulus ($${E}_{3}{\prime}$$) has a trend similar to that of in-pane storage modulus ($${E}_{1}{\prime}$$) which means the width of the yarn ($${S}_{Ff}{t}_{f}$$) significantly influences out–of–plane storage modulus ($${E}_{3}{\prime}$$) than the yarn thickness ($${t}_{f}$$). In Fig. 17f, the variation of the out-of-plane loss factor ($${\eta }_{3}$$) shows that when yarn width ($${S}_{Ff}{t}_{f}$$) increases out-of-plane loss factor ($${\eta }_{3}$$) degrades. The variation in yarn thickness ($${t}_{f})$$ has a negligible effect on the out-of-plane loss factor ($${\eta }_{3}$$).

Variations of in-plane shear storage modulus ($${G}_{12}{\prime}$$) in Fig. 17g and out-of-plane shear storage modulus ($${G}_{23}{\prime}$$) in Fig. 17i indicate that they both improve with yarn width ($${S}_{Ff}{t}_{f}$$) at lower values of yarn thickness ($${t}_{f})$$ but these properties ($${G}_{12}{\prime}$$ and $${G}_{23}{\prime}$$) deteriorate with yarn thickness ($${S}_{Ff}{t}_{f}$$) at higher values of yarn thickness ($${t}_{f}$$). Figure 17h shows the variation of in-plane shear loss factor ($${\eta }_{12}$$) which suggests that the yarn width ($${S}_{Ff}{t}_{f}$$) and yarn thickness ($${t}_{f}$$) deteriorates the in-plane shear loss factor ($${\eta }_{12}$$) which means the proposed hybrid nanocomposite system will behave more glassy when it is cyclically excited in shear mode 1–2. Out-of-plane shear loss factor ($${\eta }_{23}$$) in Fig. 17j depicts that for a higher value of yarn thickness ($${t}_{f})$$, out-of-plane shear loss factor ($${\eta }_{23}$$) improves with an increase in yarn width ($${S}_{Ff}{t}_{f}$$).

#### Effects of the gap between two yarns on the viscoelastic properties

**Fig. 18:**
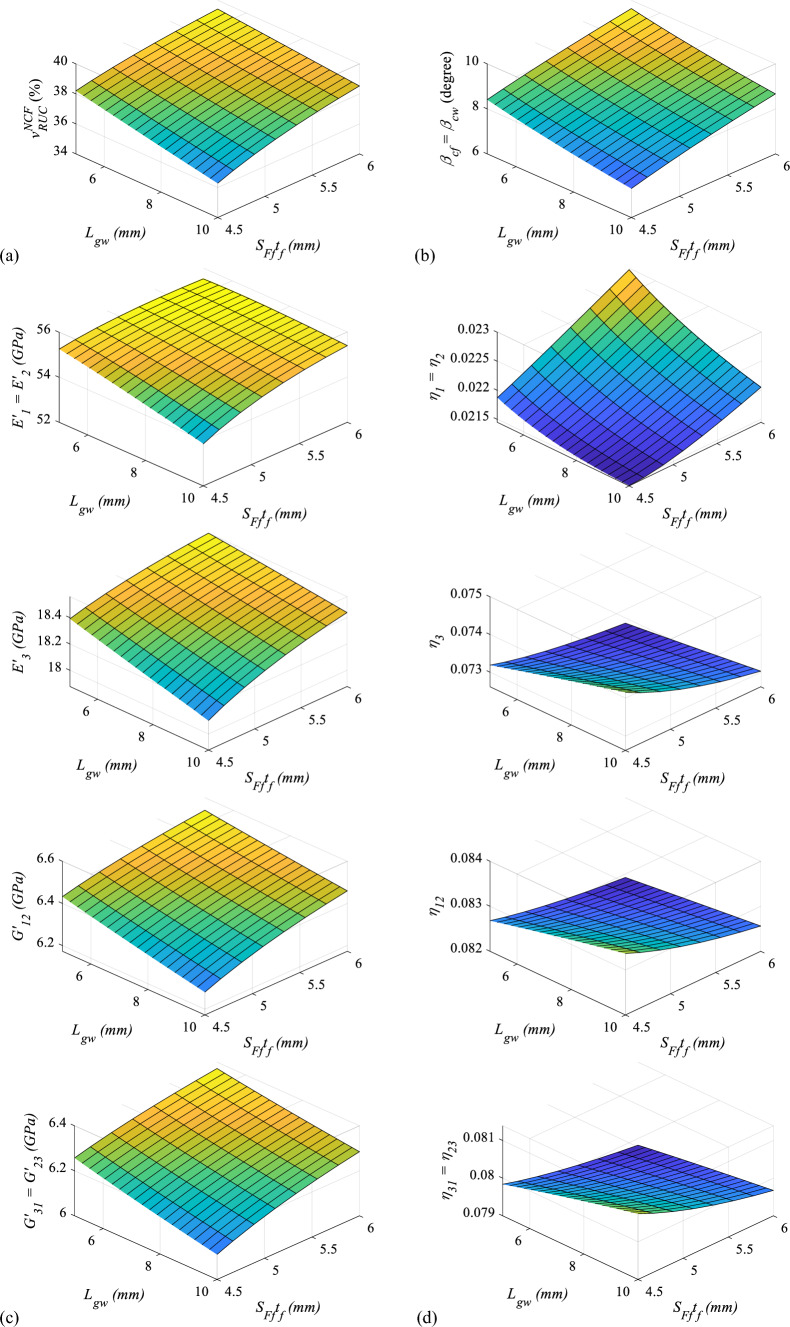
Variation of the viscoelastic properties with ${L}_{gw}$ and ${S}_{Ff}{t}_{f}$

Variation of viscoelastic properties of proposed multiscale woven fabric hybrid nanocomposite for various gap (${L}_{gw}$) and yarn width (${S}_{Ff}{t}_{f}$) are shown in Fig. 18, where Fig. 18a, b demonstrate the change in the volume fraction of fibre in $RUC$ (${v}_{RUC}^{NCF}$) and yarn crimp angle (${\beta }_{cf}$) with regard to gap (${L}_{gw}$) and yarn width (${S}_{Ff}{t}_{f}$). Figure 18a shows that ${v}_{RUC}^{NCF}$ decreases with gap (${L}_{gw}$) regardless of yarn width (${S}_{Ff}{t}_{f}$). Figure 18a shows that when the width of the yarn (${S}_{Ff}{t}_{f}$) increase, ${v}_{RUC}^{NCF}$ improves as well, irrespective of the value of gap (${L}_{gw}$). Similarly, the change of crimp angle (${\beta }_{cf}$) in Fig. 18b clarifies that it reduces with the increase of gap (${L}_{gw}$), irrespective of the value of width if the yarn (${S}_{Ff}{t}_{f}$) which can also be realized when section e-f-g-h in Fig. 6 is analyzed.

Figure 18c shows the variation of in-plane and out-of-plane storage moduli (${E}_{1}{\prime}$, ${E}_{3}{\prime}$, ${G}_{12}{\prime}$, and ${G}_{23}{\prime}$) with respect to gap (${L}_{gw}$) and yarn width (${S}_{Ff}{t}_{f}$). It can be depicted from Fig. 18c that all these properties decrease with an increase in the gap (${L}_{gw}$) however, increase with an increase in yarn width (${S}_{Ff}{t}_{f}$). The gap (${L}_{gw}$) reduces ${v}_{RUC}^{NCF}$ which causes a reduction in the storage moduli (viz. ${E}_{1}{\prime}$, ${E}_{3}{\prime}$, ${G}_{12}{\prime}$, and ${G}_{23}{\prime}$). In-pane loss factors (${\eta }_{1}$) is deteriorating with the gap (${L}_{gw}$) whereas other loss factors (viz. ${\eta }_{3}$, ${\eta }_{12}$, and ${\eta }_{23}$) in Fig. 18d indicates that all of them improves with gap (${L}_{gw}$) irrespective of the yarn width (${S}_{Ff}{t}_{f}$) suggesting that these loss factors (${\eta }_{3}$, ${\eta }_{12}$, and ${\eta }_{23}$) have an inverse trend to that of ${v}_{RUC}^{NCF}$

#### Effects of dissimilar yarns thickness on the viscoelastic properties

**Fig. 19:**
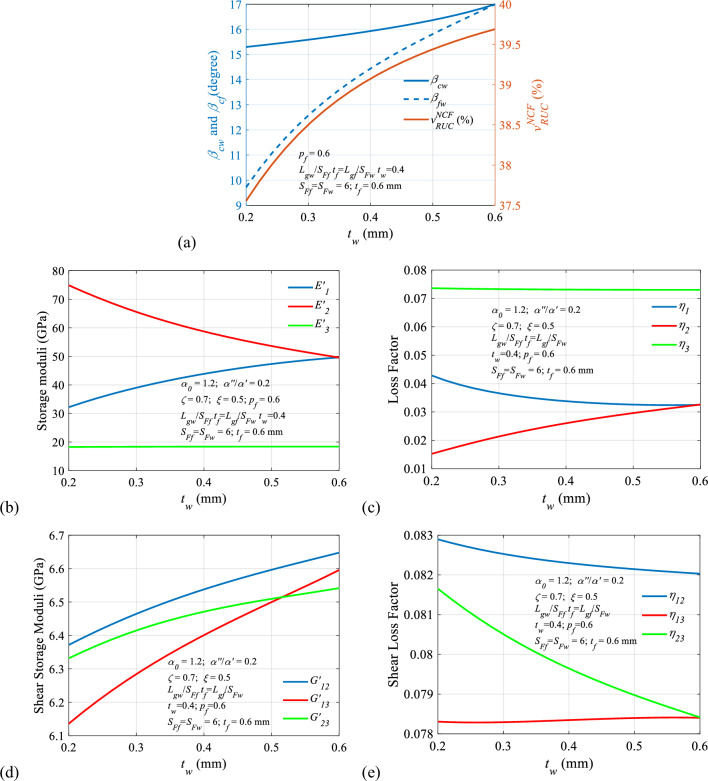
Variation of the viscoelastic properties with ${t}_{f}$

In order to study the effect of dissimilar yarn thickness on the viscoelastic properties, the thickness of warp yarn (${t}_{w}$) is varied, keeping the thickness of the fill yarn constant to assess the viscoelastic property of the multi-scale composite material system. Figure 19a depicts the variation of the volume fraction of fibre in $RUC$ (${v}_{RUC}^{NCF}$) and yarn crimp angles (${\beta }_{cf}$ and ${\beta }_{cw}$) with regard to yarn thickness (${t}_{f}$). It can be observed that both the fraction of fibre (${v}_{RUC}^{NCF}$) and crimp angles (${\beta }_{cf}$ and ${\beta }_{cw}$) increase with the increase in thickness (${t}_{w}$), which is quite apparent. It is further observed that the effect of warp thickness (${t}_{w}$) on fill yarn crimp angle (${\beta }_{cf}$) is more than that of warp yarn crimp angle (${\beta }_{cw}$). Again, the trend of volume fraction of fibre in $RUC$ (${v}_{RUC}^{NCF}$) has more semblance to the trend of fill yarn crimp angle (${\beta }_{cf}$). Figure 19b depicts the variation of longitudinal (${E}_{1}{\prime}$ and ${E}_{2}{\prime}$) and transverse (${E}_{3}{\prime}$) storage modulus with thickness (${t}_{w}$). It is worth noticing that the ${E}_{1}{\prime}$ Increase with an increase of ${t}_{w}$. On the other hand, ${t}_{w}$ has a negative impact on ${E}_{2}{\prime}$ until ${t}_{w}$< ${t}_{f}$. Warp yarn thickness does not affect transverse (${E}_{3}{\prime}$) storage modulus. Figure 19c focuses on the variation of longitudinal (${\eta }_{1}{\text{and}} {\eta }_{2}$) and transverse loss factors (${\eta }_{3}$) with thickness (${t}_{w}$). It can be observed from the figure that the longitudinal loss factor (${\eta }_{1}$) decrease with an increase in warp yarn thickness (${t}_{w}$). On the other hand, the longitudinal loss factor ${\eta }_{2}$ increase with the increase in warp yarn thickness (${t}_{w}$) until ${t}_{w}$< ${t}_{f}$. The transverse loss factor (${\eta }_{3}$) is uninfluenced with warp yarn thickness (${t}_{w}$). As depicted in Fig. 19d, all the shear storage moduli (${G}_{12}{\prime}$, ${G}_{13}{\prime}$ and ${G}_{23}{\prime}$) improve with an increase in warp yarn thickness (${t}_{w}$) whereas Fig. 19e, suggests that shear loss factors ${\eta }_{12}$, and ${\eta }_{23}$ deteriorate with an increase in warp yarn thickness (${t}_{w}$) whereas ${\eta }_{13}$ show slight improvement with warp yarn thickness (${t}_{w}$). It is worth mentioning that the variation in the thickness of warp and fill yarn will result in making the material system anisotropic.

## Performance evaluation test

**Fig. 20:**
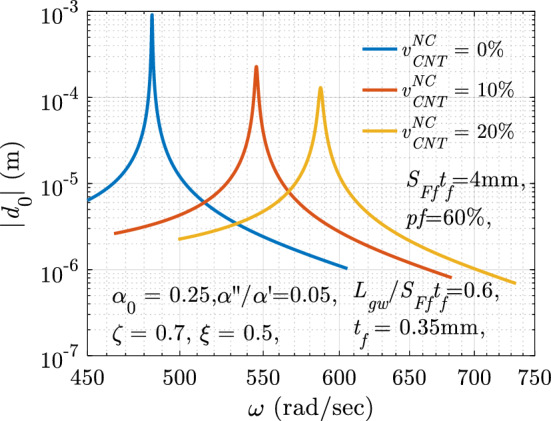
Frequency responses of the proposed hybrid spherical shell panel ($R/a$ =10 and $a/h$ =100) for various ${v}_{NC}^{CNT}$

**Fig. 21:**
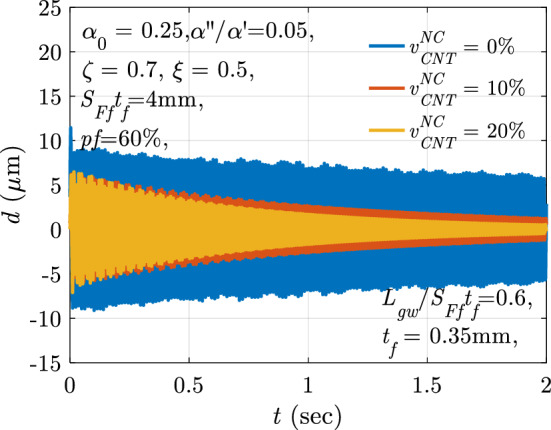
Impulse responses of the proposed hybrid spherical shell panel ($R/a$ =10 and $a/h$ =100) for various ${v}_{NC}^{CNT}$

In order to evaluate the performance of the proposed composite, a finite element based vibration test has been conducted on spherical laminate with the stacking sequence of [0/-45/45/90]_2 s_ and simply supported boundary conditions as taken in Ref. [95, 96] after proper validation and convergence study of the shell finite element formulations (Ref. [66, 97]) as implemented by Swain and Roy [66]. A shell described here is the most versatile structural element that can consider such material properties. The versatility of the shell is such that it can approximate a plate with a higher radius of curvature. It can behave as a beam if one edge is comparatively smaller than another. To highlight the versatility of the micromechanics and for brevity, only one type of geometry of the shell panel with only simply supported boundary condition is considered in this study. The required matrices used for the performance evaluation study are also mentioned in Appendix E. In the current performance evaluation ${v}_{NC}^{CNT}$ is varied as 0%, 10% and 20% keeping all the parameters constants as shown in Frequency responses plot in Fig. 20. It is evident from Fig. 20 as ${v}_{NC}^{CNT}$ is increased, the absolute amplitude of the shell mid-surface ($\left|{d}_{0}\right|$) is decreased, whereas the resonant frequency is shifted to higher values desired for a wide range of applications. The results of the modal analysis of such shell panels are shown in Table 4. It is depicted from Table 4 that the first natural frequency and the first modal loss factors also improved due to in collusion of CNTs in the polymer phase. It is clear that the CNTs have improved the natural frequency by 12.66% and 21.24% and modal loss factors by 255.61% and 392.14% by increasing of ${v}_{NC}^{CNT}$ by 10% and 20% respectively. The impulse response shown in Fig. 21 shows a similar implication of improving the damping of the spherical shell panel. It is evident from the figure that the response of the panel with the highest CNTs content damps more effectively when compared to shell panels with lesser CNTs content.

**Table 4:** Comparison of first natural frequency and loss factor for shell panel with $R/a$ =10 and $a/h$ =100

Parameters		${\omega }_{1}$	${\eta }_{1}$
${v}_{NC}^{CNT}$(% age)	0	484.39	08.574 × 10^–04^
	10	545.71	27.918 × 10^–04^
	20	587.26	42.196 × 10^–04^

## Conclusions

This study introduces a novel micromechanical framework for evaluating the viscoelastic properties of a two-dimensional (2D) woven hybrid composite reinforced with multiscale carbon nanotubes (CNTs). Initially, a comprehensive description of the constructional features of the 2D woven trans-scale composite material system is provided. The mathematical modeling of the material properties of each constituent phase or building block is established to evaluate the homogenized viscoelastic properties of the proposed composite material system based on the nature of the constructional feature. In order to emphasize the originality of this research, the viscoelastic properties of the modified matrix are evaluated by using the Tanaka (MT) micromechanics method in conjunction with the weak viscoelastic interphase (WI) theory. The CNTs are assumed to be randomly orientated inside the polymer phase. The mathematical frameworks for the viscoelastic features of yarns are established using the strength of the material (SOM) approach. In contrast, the viscoelastic properties of the representative unit cell (RUC) are determined using the unit cell method (UCM). Variations in the carbon nanotube (CNT) content, interface conditions, agglomeration, carbon fiber volume fraction, excitation frequency, and temperature have yielded diverse numerical outcomes. This study also investigates the impact of geometrical characteristics, such as yarn thickness, breadth, and gap length, on the viscoelasticity of composite material systems. The study has shown that not only all the storage moduli but also all the material loss factors improved with CNT content. On the other hand, the aggregation deteriorated the storage modulus. The result suggests that the deterioration of all the storage and loss factors is significant at higher CNT content. Again, it is also verified that the aspect ratio of CNT helps improve the storage modulus. However, the loss factors improved first with the aspect ratio and became unaffected by a further increase in the aspect ratio. The weak interphase deteriorated the storage modulus but improved the loss factors. The yarn geometry is essential in determining the viscoelastic property of the material system. It is observed that the yarn thickness has negatively impacted the longitudinal and transverse storage modulus but improved all the shear storage modulus. All the loss factors except the out-of-plane shear loss factor declined with increased yarn width. Out-of-plane shear loss factor showed a mixed trend; at a lower value of yarn thickness, it deteriorated and improved at a higher value of yarn thickness. Gap has a negative effect on all the storage and inplane loss factors. On the other hand, out-of-plane loss factors and all shear loss factors improved with gap length. The present investigation also examines the matter of anisotropic viscoelastic properties that arise as a consequence of employing yarns of unequal thicknesses. The findings of this micromechanical research offer significant insights into the viscoelastic characteristics of the proposed composite material system and indicate its potential utility in the field of vibration damping. In order to showcase the practical implementation of newly developed micromechanics in the analysis of vibration, a significant contribution is made by comprehensive numerical experiments on a shell panel. In the frequency response and transient response analysis, the findings indicate a notable decrease in vibration amplitudes when compared to conventional composite materials. In order to prioritize the examination of micromechanical behavior in connection to dynamic response, this study exclusively considers linear strain displacement relationships for brevity. These observations have the potential to guide future investigations and advancements in the realm of composite materials. Numerical performance study reveals that these multiscale hybrid composites can be used in the application of vibration reduction.

## Data Availability

The authors declare that the data supporting the findings of this study are available within the paper.

## Appendix A

Eshelby’s tensor is given by [98]

A1 $${S}_{ijkl}=\frac{1}{8\pi }{C}_{pqmn}^{P}\left({G}_{ipjq}+{G}_{jpiq}\right)$$

$${\text{G}}_{1111}=\frac{\pi }{2}\underset{0}{\overset{1}{\int }}\Delta \left(1-{x}^{2}\right)\left[\left\{\widehat{f}\left(1-{x}^{2}\right)+\widehat{h}{\widehat{\rho }}^{2}{x}^{2}\right\}\left\{\left(3\widehat{e}+\widehat{d}\right)\left(1-{x}^{2}\right)+4\widehat{f}{\widehat{\rho }}^{2}{x}^{2}\right\}-{\widehat{g}}^{2}{\widehat{\rho }}^{2}{x}^{2}\left(1-{x}^{2}\right)\right]dx$$

$${G}_{1122}=\frac{\pi }{2}{\int }_{0}^{1}\Delta \left(1-{x}^{2}\right)\left[\left\{\widehat{f}\left(1-{x}^{2}\right)+\widehat{h}{\widehat{\rho }}^{2}{x}^{2}\right\}\left\{\left(\widehat{e}+3\widehat{d}\right)\left(1-{x}^{2}\right)+4\widehat{f}{\widehat{\rho }}^{2}{x}^{2}\right\}-3{\widehat{g}}^{2}{\widehat{\rho }}^{2}{x}^{2}\left(1-{x}^{2}\right)\right]dx$$

$${G}_{1133}=2\pi \underset{0}{\overset{1}{\int }}\Delta {\widehat{\rho }}^{2}{x}^{2}\left[\left\{\left(\widehat{d}+\widehat{e}\right)\left(1-{x}^{2}\right)+2\widehat{f}{\widehat{\rho }}^{2}{x}^{2}\right\}\left\{\widehat{f}\left(1-{x}^{2}\right)+\widehat{h}{\widehat{\rho }}^{2}{x}^{2}\right\}-{\widehat{g}}^{2}{\widehat{\rho }}^{2}{x}^{2}\left(1-{x}^{2}\right)\right]dx$$

$${G}_{1212}=\frac{\pi }{2}{\int }_{0}^{1}\Delta {\left(1-{x}^{2}\right)}^{2}\left[{\widehat{g}}^{2}{\widehat{\rho }}^{2}{x}^{2}-\left(\widehat{d}-\widehat{e}\right)\left\{\widehat{f}\left(1-{x}^{2}\right)+\widehat{h}{\widehat{\rho }}^{2}{x}^{2}\right\}\right]dx$$

$${G}_{1313}=2\pi \underset{0}{\overset{1}{\int }}\Delta \widehat{g}{\widehat{\rho }}^{2}{x}^{2}\left(1-{x}^{2}\right)\left\{\widehat{e}\left(1-{x}^{2}\right)+\widehat{f}{\widehat{\rho }}^{2}{x}^{2}\right\}dx$$

$${G}_{3311}=2\pi \underset{0}{\overset{1}{\int }}\Delta \left(1-{x}^{2}\right)\left\{\widehat{d}\left(1-{x}^{2}\right)+\widehat{f}{\widehat{\rho }}^{2}{x}^{2}\right\}\left\{\widehat{e}\left(1-{x}^{2}\right)+\widehat{f}{\widehat{\rho }}^{2}{x}^{2}\right\}dx$$

$${G}_{3333}=4\pi \underset{0}{\overset{1}{\int }}\Delta {\widehat{\rho }}^{2}{x}^{2}\left\{\widehat{d}\left(1-{x}^{2}\right)+\widehat{f}{\widehat{\rho }}^{2}{x}^{2}\right\}\left\{\widehat{e}\left(1-{x}^{2}\right)+\widehat{f}{\widehat{\rho }}^{2}{x}^{2}\right\}dx$$

$${\text{G}}_{2222}{= \text{ G} }_{1111}$$

$${\text{G}}_{2211}{= \text{ G} }_{1122}$$

$${\text{G}}_{2233}{= \text{ G} }_{1133}$$

$${\text{G}}_{3322}{= \text{ } {\text{G}}}_{3311}$$

$${\text{G}}_{2323}{= \text{ G} }_{1313}$$

and

$$\widehat{d}={C}_{1111}^{P}$$

$$\widehat{e}\text{=}\frac{{C}_{1111}^{P}-{C}_{1122}^{P}}{2}$$

$$\widehat{f}={C}_{2323}^{P}$$

$$\widehat{g}={C}_{1133}^{P}+{C}_{2323}^{P}$$

$$\widehat{h}={C}_{3333}^{P}$$

$$\widehat{\rho }=\frac{1}{\widehat{\alpha }}$$

$${\Delta }^{-1}=\left\{\widehat{e}\left(1-{x}^{2}\right)+\widehat{f}{\widehat{\rho }}^{2}{x}^{2}\right\}\left[\left\{\widehat{d}\left(1-{x}^{2}\right)+\widehat{f}{\widehat{\rho }}^{2}{x}^{2}\right\}\left\{\widehat{f}\left(1-{x}^{2}\right)+\widehat{h}{\widehat{\rho }}^{2}{x}^{2}\right\}-{\widehat{g}}^{2}{\widehat{\rho }}^{2}{x}^{2}\left(1-{x}^{2}\right)\right]$$

For $\widehat{\alpha }=\infty$, an explicit expressions for the nonzero element of the Eshelby’s tensor reported by Li and Dunn [99] are.

$${S}_{1111}={S}_{2222}=\frac{5{C}_{1111}^{P}+{C}_{1122}^{P}}{8{C}_{1111}^{P}}$$

$${S}_{1122}={S}_{2211}=\frac{3{C}_{1122}^{P}-{C}_{1111}^{P}}{8{C}_{1111}^{P}}$$

$${S}_{1212}={S}_{1221}={S}_{2112}={S}_{2121}=\frac{3{C}_{1111}^{P}-{C}_{1122}^{P}}{8{C}_{1111}^{P}}$$

$${S}_{1133}={S}_{2233}=\frac{{C}_{1133}^{P}}{2{C}_{1111}^{P}}$$

$${S}_{2323}={S}_{2332}={S}_{3223}={S}_{3232}={S}_{1313}={S}_{1331}={S}_{3113}=\frac{1}{4}$$

## Appendix B

Parameters associated with weak interface

B1 $${P}_{ijkl}=\frac{3}{16\pi }\underset{0}{\overset{\pi }{\int }}\left[\underset{0}{\overset{2\pi }{\int }}\frac{{\delta }_{ik}{\widehat{n}}_{j}{\widehat{n}}_{l}+{\delta }_{jk}{\widehat{n}}_{i}{\widehat{n}}_{l}+{\delta }_{il}{\widehat{n}}_{k}{\widehat{n}}_{j}+{\delta }_{jl}{\widehat{n}}_{k}{\widehat{n}}_{i}}{n}d\theta \right]{\text{sin}}\phi d\phi$$

B2 $${Q}_{ijkl}=\frac{3}{4\pi }\underset{0}{\overset{\pi }{\int }}\left[\underset{0}{\overset{2\pi }{\int }}\frac{{\widehat{n}}_{i}{\widehat{n}}_{j}{\widehat{n}}_{k}{\widehat{n}}_{l}}{{n}^{3}}d\theta \right]{\text{sin}}\phi d\phi$$

$$n=\sqrt{{\widehat{n}}_{i}{\widehat{n}}_{i}}$$

$${\widehat{n}}_{i}={\left(\begin{array}{ccc}\frac{{\text{sin}}\phi {\text{cos}}\theta }{{a}_{1}}& \frac{{\text{sin}}\phi {\text{sin}}\theta }{{a}_{2}}& \frac{{\text{cos}}\phi }{{a}_{3}}\end{array}\right)}^{T}$$

For cylindrical inclusions ${a}_{1}={a}_{2}=a$ and ${a}_{3}\to \infty$. Hence equation above reduces to.

$${P}_{1111}={P}_{2222}=4{P}_{2323}=4{P}_{1313}=2{P}_{1212}=\frac{3\pi }{8a}$$

$${Q}_{1111}={Q}_{2222}=3{Q}_{1122}=3{Q}_{2211}=3{Q}_{1212}=\frac{9\pi }{32a}$$

With other terms being zeros.

## Appendix C

C1 $$\left[{V}_{1}\right]={\left[\left[{V}_{3}\right]+\left[{V}_{4}\right]{\left[{C}_{4}\right]}^{-1}\left[{C}_{3}\right]\right]}^{-1}\left[{V}_{2}\right]={\left[\left[{V}_{4}\right]+\left[{V}_{3}\right]{\left[{C}_{3}\right]}^{-1}\left[{C}_{4}\right]\right]}^{-1};$$

C2 $$\left[{C}_{1}\right]={v}_{NCF}^{F}\left[\begin{array}{cccccc}{c}_{11}^{F}& {c}_{12}^{F}& {c}_{13}^{F}& 0& 0& 0\\ 0& 0& 0& 0& 0& 0\\ 0& 0& 0& 0& 0& 0\\ 0& 0& 0& 0& 0& 0\\ 0& 0& 0& 0& 0& 0\\ 0& 0& 0& 0& 0& 0\end{array}\right]$$

C3 $$\left[{C}_{2}\right]=\left[\begin{array}{cccccc}{v}_{NCF}^{NC}{c}_{11}^{NC}& {v}_{NCF}^{NC}{c}_{12}^{NC}& {v}_{NCF}^{NC}{c}_{13}^{NC}& 0& 0& 0\\ {c}_{12}^{NC}& {c}_{22}^{NC}& {c}_{23}^{NC}& 0& 0& 0\\ {c}_{13}^{NC}& {c}_{23}^{NC}& {c}_{33}^{NC}& 0& 0& 0\\ 0& 0& 0& {c}_{44}^{NC}& 0& 0\\ 0& 0& 0& 0& {c}_{55}^{NC}& 0\\ 0& 0& 0& 0& 0& {c}_{66}^{NC}\end{array}\right];$$

C4 $$\left[{V}_{3}\right]=\left[\begin{array}{cccccc}0& 0& 0& 0& 0& 0\\ 0& {v}_{NCF}^{F}& 0& 0& 0& 0\\ 0& 0& {v}_{NCF}^{F}& 0& 0& 0\\ 0& 0& 0& {v}_{NCF}^{F}& 0& 0\\ 0& 0& 0& 0& {v}_{NCF}^{F}& 0\\ 0& 0& 0& 0& 0& {v}_{NCF}^{F}\end{array}\right];$$

C4 $$\left[{V}_{4}\right]=\left[\begin{array}{cccccc}1& 0& 0& 0& 0& 0\\ 0& {v}_{NCF}^{NC}& 0& 0& 0& 0\\ 0& 0& {v}_{NCF}^{NC}& 0& 0& 0\\ 0& 0& 0& {v}_{NCF}^{NC}& 0& 0\\ 0& 0& 0& 0& {v}_{NCF}^{NC}& 0\\ 0& 0& 0& 0& 0& {v}_{NCF}^{NC}\end{array}\right];$$

$$\left[{C}_{3}\right]=\left[\begin{array}{cccccc}1& 0& 0& 0& 0& 0\\ {c}_{12}^{F}& {c}_{22}^{F}& {c}_{23}^{F}& 0& 0& 0\\ {c}_{13}^{F}& {c}_{23}^{F}& {c}_{33}^{F}& 0& 0& 0\\ 0& 0& 0& {c}_{44}^{F}& 0& 0\\ 0& 0& 0& 0& {c}_{55}^{F}& 0\\ 0& 0& 0& 0& 0& {c}_{66}^{F}\end{array}\right];$$

C5 $$\left[{C}_{4}\right]=\left[\begin{array}{cccccc}1& 0& 0& 0& 0& 0\\ {c}_{12}^{NC}& {c}_{22}^{NC}& {c}_{23}^{NC}& 0& 0& 0\\ {c}_{13}^{NC}& {c}_{23}^{NC}& {c}_{33}^{NC}& 0& 0& 0\\ 0& 0& 0& {c}_{44}^{NC}& 0& 0\\ 0& 0& 0& 0& {c}_{55}^{NC}& 0\\ 0& 0& 0& 0& 0& {c}_{66}^{NC}\end{array}\right]$$

## Appendix D

D1 $$\begin{gathered} A_{{\bar{w}}} = \frac{{3\beta _{{cw}} }}{8} + \frac{{\sin \left( {2\beta _{{cw}} } \right)}}{4} + \frac{{\sin \left( {4\beta _{{cw}} } \right)}}{{32}};\quad B_{{\bar{w}}} = \frac{{3\beta _{{cw}} }}{8} - \frac{{\sin \left( {2\beta _{{cw}} } \right)}}{4} + \frac{{\sin \left( {4\beta _{{cw}} } \right)}}{{32}} \hfill \\ C_{{\bar{w}}} = \frac{{\beta _{{cw}} }}{8} - \frac{{\sin \left( {4\beta _{{cw}} } \right)}}{{32}};\quad D_{{\bar{w}}} = \frac{{\beta _{{cw}} }}{2} + \frac{{\sin \left( {2\beta _{{cw}} } \right)}}{4};\quad E_{{\bar{w}}} = \frac{{\beta _{{cw}} }}{2} - \frac{{\sin \left( {2\beta _{{cw}} } \right)}}{4} \hfill \\ F_{{\bar{w}}} = \frac{{\cos ^{4} \left( {\beta _{{cw}} } \right)}}{2} - \frac{1}{2};\quad G_{{\bar{w}}} = \left( {\cos ^{2} \left( {\beta _{{cw}} } \right) - 1} \right)^{2} ;\quad H_{{\bar{w}}} = \frac{{F_{{\bar{w}}} }}{2} + \frac{{G_{{\bar{w}}} }}{4} \hfill \\ I_{{\bar{w}}} = \frac{{\sin ^{2} \left( {\beta _{{cw}} } \right)}}{2};\quad J_{{\bar{w}}} = \frac{{\beta _{{cw}} }}{2} - \frac{{\sin \left( {4\beta _{{cw}} } \right)}}{8};\quad K_{{\bar{w}}} = \frac{{\beta _{{cw}} }}{2} + \frac{{\sin \left( {4\beta _{{cw}} } \right)}}{8} \hfill \\ \end{gathered}$$

## Appendix E

The equation of motion can be expressed as follows:

E1 $$\left[{M}_{uu}^{e}\right]\left\{{\ddot{d}}^{e}\right\}+\left[{K}_{uu}^{e}\right]\left\{{d}^{e}\right\}=\left\{{f}^{e}\right\}$$

In its final form, the mass matrix can be stated as follows:

E2 $$\left[{M}_{uu}^{e}\right]=\underset{-1}{\overset{1}{\int }}\underset{-1}{\overset{1}{\int }}\underset{-h/2}{\overset{h/2}{\int }}\rho {\left[N\right]}^{T}\left[N\right]dz\left|J\right|d\xi d\eta$$

The matrix of element structural stiffness can be calculated as follows [97]:

E3 $$\left[{K}_{uu}^{e}\right]=\underset{V}{\int }{\left[{B}_{u}^{e}\right]}^{T}\left[C\right]\left[{B}_{u}^{e}\right]dV$$

$$\left[{K}_{uu}^{e}\right]=\left[{K}_{bb}^{e}\right]+\left[{K}_{ss}^{e}\right]$$

$$\left[{B}_{u}^{e}\right]=\left[\begin{array}{cc}\left[{B}_{b}^{e}\right]& \left[0\right]\\ \left[0\right]& \left[{B}_{s}^{e}\right]\end{array}\right]$$

$$\left[C\right]=\left[\begin{array}{cc}\left[{D}_{b}\right]& \left[0\right]\\ \left[0\right]& \left[{D}_{s}\right]\end{array}\right]$$

The element in a laminated composite structure's plane/bending stiffness matrix $\left[{K}_{bb}^{e}\right]$ can be calculated as follows.

$$\left[{K}_{bb}^{e}\right]=\underset{V}{\int }{\left[{B}_{b}^{e}\right]}^{T}\left[{D}_{b}\right]\left[{B}_{b}^{e}\right]dV=\underset{\Omega }{\int }{\left[{B}_{b}^{e}\right]}^{T}\left[\begin{array}{cc}\left[A\right]& \left[B\right]\\ \left[B\right]& \left[D\right]\end{array}\right]\left[{B}_{b}^{e}\right]d\Omega$$

The transverse shear stiffness matrix $\left[{K}_{ss}^{e}\right]$ of an element can be represented as follows:

$$\left[{K}_{ss}^{e}\right]=\underset{\Omega }{\int }{\left[{B}_{s}^{e}\right]}^{T}\left[{D}_{s}\right]\left[{B}_{s}^{e}\right]d\Omega$$

The element external force vector $\left\{{f}^{e}\right\}$ can be obtained if the external load is dispersed over the surface with a magnitude of ${f}_{s}^{e}$ by using the relation:

E4 $$\left\{{f}^{e}\right\}=\underset{A}{\int }{\left[N\right]}^{T}\left\{{f}_{s}^{e}\left(x,y\right)\right\}dA$$

The strain displacement relation can be found by [95]

E5 $$\left[{B}_{b}^{e}\right]=\sum\left[\begin{array}{ccccc} \, \frac{\partial {N}_{i}}{\partial {\alpha }_{1}}& \, \frac{{N}_{i}}{{A}_{1}{A}_{2}}\frac{\partial {A}_{1}}{\partial {\alpha }_{2}} \, & \frac{{N}_{i}}{{R}_{1}}& \text{ 0}& \, 0\\ \, \frac{{N}_{i}}{{A}_{1}{A}_{2}}\frac{\partial {A}_{2}}{\partial {\alpha }_{1}}& \, \frac{1}{{A}_{2}}\frac{\partial {N}_{i}}{\partial {\alpha }_{2}}& \frac{{N}_{i}}{{R}_{2}}& \, 0& \, 0\\ \, \frac{1}{{A}_{2}}\frac{\partial {N}_{i}}{\partial {\alpha }_{2}}-\frac{{N}_{i}}{{A}_{1}{A}_{2}}\frac{\partial {A}_{1}}{\partial {\alpha }_{2}}& \, \frac{1}{{A}_{1}}\frac{\partial {N}_{i}}{\partial {\alpha }_{1}}-\frac{{N}_{i}}{{A}_{1}{A}_{2}}\frac{\partial {A}_{2}}{\partial {\alpha }_{1}}& \frac{2{N}_{i}}{{R}_{12}}& \, 0& \, 0\\ -\frac{1}{2}\frac{1}{{R}_{12}}\left(\frac{1}{{A}_{2}}\frac{\partial {N}_{i}}{\partial {\alpha }_{2}}+\frac{{N}_{i}}{{A}_{1}{A}_{2}}\frac{\partial {A}_{1}}{\partial {\alpha }_{2}}\right)& \, \frac{1}{2}\frac{1}{{R}_{12}}\left(\frac{1}{{A}_{1}}\frac{\partial {N}_{i}}{\partial {\alpha }_{1}}+\frac{{N}_{i}}{{A}_{1}{A}_{2}}\frac{\partial {A}_{2}}{\partial {\alpha }_{1}}\right)& 0& \, \frac{1}{{A}_{1}}\frac{\partial {N}_{i}}{\partial {\alpha }_{1}}& \, \frac{{N}_{i}}{{A}_{1}{A}_{2}}\frac{\partial {A}_{1}}{\partial {\alpha }_{2}}\\ \, \frac{1}{2}\frac{1}{{R}_{12}}\left(\frac{1}{{A}_{2}}\frac{\partial {N}_{i}}{\partial {\alpha }_{2}}+\frac{{N}_{i}}{{A}_{1}{A}_{2}}\frac{\partial {A}_{1}}{\partial {\alpha }_{2}}\right)& -\frac{1}{2}\frac{1}{{R}_{12}}\left(\frac{1}{{A}_{1}}\frac{\partial {N}_{i}}{\partial {\alpha }_{1}}+\frac{{N}_{i}}{{A}_{1}{A}_{2}}\frac{\partial {A}_{2}}{\partial {\alpha }_{1}}\right)& 0& \, \frac{{N}_{i}}{{A}_{1}{A}_{2}}\frac{\partial {A}_{2}}{\partial {\alpha }_{1}}& \, \frac{1}{{A}_{2}}\frac{\partial {N}_{i}}{\partial {\alpha }_{2}}\\ \, {C}_{0}\left(\frac{1}{{A}_{2}}\frac{\partial {N}_{i}}{\partial {\alpha }_{2}}+\frac{{N}_{i}}{{A}_{1}{A}_{2}}\frac{\partial {A}_{1}}{\partial {\alpha }_{2}}\right)& \, -{C}_{0}\left(\frac{1}{{A}_{1}}\frac{\partial {N}_{i}}{\partial {\alpha }_{1}}+\frac{{N}_{i}}{{A}_{1}{A}_{2}}\frac{\partial {A}_{2}}{\partial {\alpha }_{1}}\right)& 0& \frac{1}{{A}_{2}}\frac{\partial {N}_{i}}{\partial {\alpha }_{2}}-\frac{{N}_{i}}{{A}_{1}{A}_{2}}\frac{\partial {A}_{1}}{\partial {\alpha }_{2}}& \frac{1}{{A}_{1}}\frac{\partial {N}_{i}}{\partial {\alpha }_{1}}-\frac{{N}_{i}}{{A}_{1}{A}_{2}}\frac{\partial {A}_{2}}{\partial {\alpha }_{1}}\end{array}\right]$$

where, $${C}_{0}=\frac{1}{2}\left(\frac{1}{{R}_{1}}-\frac{1}{{R}_{2}}\right)$$

E6 $$\left[{B}_{s}^{e}\right]=\sum\left[\begin{array}{ccccc}-\frac{{N}_{i}}{{R}_{12}}& -\frac{{N}_{i}}{{R}_{2}}& \frac{1}{{A}_{2}}\frac{\partial {N}_{i}}{\partial {\alpha }_{2}}& \, 0& {N}_{i}\\ -\frac{{N}_{i}}{{R}_{1}}& -\frac{{N}_{i}}{{R}_{12}}& \frac{1}{{A}_{1}}\frac{\partial {N}_{i}}{\partial {\alpha }_{1}}& {N}_{i}& \, 0\end{array}\right]$$

where,

$$\begin{array}{c}{N}_{1}=\frac{1}{4}(1-\xi )(1-\eta )(-1-\xi -\eta )\\ {N}_{2}=\frac{1}{2}(1-{\xi }^{2})(1-\eta )\\ {N}_{3}=\frac{1}{4}(1+\xi )(1-\eta )(-1+\xi -\eta )\\ {N}_{4}=\frac{1}{2}(1+\xi )(1-{\eta }^{2})\\ {N}_{5}=\frac{1}{4}(1+\xi )(1+\eta )(-1+\xi +\eta )\\ {N}_{6}=\frac{1}{2}(1-{\xi }^{2})(1+\eta )\\ {N}_{7}=\frac{1}{4}(1-\xi )(1+\eta )(-1-\xi +\eta )\\ {N}_{8}=\frac{1}{2}(1-\xi )(1-{\eta }^{2})\end{array}$$

E7 $$\left[N\right]=\left[\begin{array}{ccccccccccc}{N}_{1}& 0& 0& 0& 0& ...& {N}_{n}& 0& 0& 0& 0\\ 0& {N}_{1}& 0& 0& 0& ...& 0& {N}_{n}& 0& 0& 0\\ 0& 0& {N}_{1}& 0& 0& ...& 0& 0& {N}_{n}& 0& 0\\ 0& 0& 0& {N}_{1}& 0& ...& 0& 0& 0& {N}_{n}& 0\\ 0& 0& 0& 0& {N}_{1}& ...& 0& 0& 0& 0& {N}_{n}\end{array}\right]$$

E8 $$\left[C\right]=\left[\begin{array}{cc}\left[{D}_{b}\right]& \left[0\right]\\ \left[0\right]& \left[{D}_{s}\right]\end{array}\right]=\left[\begin{array}{cccccccc}{A}_{11}& {A}_{12}& {A}_{16}& {B}_{11}& {B}_{12}& {B}_{16}& 0& 0\\ {A}_{12}& {A}_{22}& {A}_{26}& {B}_{12}& {B}_{22}& {B}_{26}& 0& 0\\ {A}_{16}& {A}_{26}& {A}_{66}& {B}_{16}& {B}_{26}& {B}_{66}& 0& 0\\ {B}_{11}& {B}_{12}& {B}_{16}& {D}_{11}& {D}_{12}& {D}_{16}& 0& 0\\ {B}_{12}& {B}_{22}& {B}_{26}& {D}_{12}& {D}_{22}& {D}_{26}& 0& 0\\ {B}_{16}& {B}_{26}& {B}_{66}& {D}_{16}& {D}_{26}& {D}_{66}& 0& 0\\ 0& 0& 0& 0& 0& 0& {A}_{44}& {A}_{45}\\ 0& 0& 0& 0& 0& 0& {A}_{45}& {A}_{55}\end{array}\right]$$

E9 $$\left(\begin{array}{ccc}{A}_{ij}& {B}_{ij}& {D}_{ij}\end{array}\right)={\int }_{-\frac{h}{2}}^{+\frac{h}{2}}\left(\begin{array}{ccc}1& z& {z}^{2}\end{array}\right)\overline{{Q }_{ij}}\text{dz;}\left({A}_{ij}^{s}\right)={\int }_{-\frac{h}{2}}^{+\frac{h}{2}}{k}_{s}\overline{{Q }_{ij}}{\text{dz}}$$

## List of symbols

CNT: Carbon-nanotube
2D: 2-Dimensional
MT: Mori-Tanaka
WI: Weak viscoelastic interphase
SOM: Strength of materials
UCM: Unit cell method
RUC: Representative unit cell
NC: Nano-composite
pf: Packing factor
RVE: Representative volume element
MWCNT: Multi-walled carbon-nanotube
CFRP: Carbon fiber reinforced polymer
GA: Genetic algorithm
$\sigma^{P}$: Stress of matrix phase
$\dot{\varepsilon }^{P}$: Strain rate of matrix phase
$C^{P}$: Stiffness of matrix phase
$E_{\infty }^{P}$, $E_{j}^{P}$, $\tau_{i}^{P}$: Coefficients of the Prony series
$\overline{\sigma }^{NC}$: Average state of stresses in the configuration of ‘a’
$\overline{\sigma }^{P}$: Average state of stresses in the configuration of ‘b’
$$\varepsilon^{ * }$$: Eigen-strain
*S*: Eshelby’s tensor
$S^{P}$: Compliance tensor for polymer material
$I$: Fourth order identity tensor
*ε*_*a*_: Average strain
$v_{NC}^{P}$: Volume fractions of matrix
$v_{NC}^{CNT}$: Volume fractions of CNTs
$A^{{{\text{ndil}}}}$: Non-dilute strain concentration tensor
$$S_{I}$$: Interface
$n$: Unit vector normal to $S_{I}$
$u_{i} (S_{I}^{ + } )$: Displacements when approached from inside of the inclusion
$u_{i} (S_{I}^{ - } )$: Displacements when approached from outside of the inclusion
$V_{NC}^{CNT}$: Total volume of CNTs
$V_{Cluster}^{CNT}$: CNTs trapped inside the cluster
$V_{P}^{CNT}$: Remaining CNT in the polymer phase outside the cluster
*ξ*: Volume fraction of the spherical cluster with respect to the RVE
$d_{cnt}$: Diameter of CNTs
$l_{cnt}$: Length of CNTs
$$\alpha$$: Interfacial compliance
$\alpha^{\prime}$: Real part of interfacial compliance
$\alpha^{\prime\prime}$: Imaginary part of interfacial compliance
${{\alpha^{\prime\prime}} \mathord{\left/ {\vphantom {{\alpha^{\prime\prime}} {\alpha^{\prime}}}} \right. \kern-0pt} {\alpha^{\prime}}}$: Interfacial loss factor
$\alpha_{0}$: Non-dimensional interfacial compliance
$v_{F}$: Volume fractions of carbon fibres
$v_{NC}$: Volume fractions of modified matrix
$r_{f} ,r_{w}$: Radius of curvature of fill and warp yarn
$t_{f} ,t_{w}$: Thickness of fill and warp yarn
$S_{Ff} ,S_{Fw}$: Shape factor of fill and warp yarn
$S_{Ff} t_{f} ,S_{Fw} t_{w}$: Width of fill and warp yarn
${\beta }_{cf}$ and ${\beta }_{cw}$: Crimp angles corresponding to fill and warp yarn
